# Exploiting the combined dynamic and geometric phases for optical vortex beam generation using metasurfaces

**DOI:** 10.1515/nanoph-2025-0004

**Published:** 2025-03-11

**Authors:** Jialong Cui, Chen Qing, Lishuang Feng, Dengke Zhang

**Affiliations:** School of Instrumentation and Optoelectronic Engineering, Beihang University, Beijing 100191, China

**Keywords:** optical vortices, dynamic phase, geometric phase, metasurface, orbital angular momentum

## Abstract

The generation of optical vortex beams is pivotal for a myriad of applications, encompassing optical tweezing, optical communications, and quantum information, among others. The metasurface-based approach has realized significant advancements in vortex production, utilizing either dynamic or geometric phases. The dynamic design exhibits indifference to the polarization state of incident light, while the geometric design is inextricably tied to it. In the study, we put forth the proposition that combining dynamic and geometric phases could unlock the potential of metasurface design in generating optical vortices. A hybrid design that harnesses the combined dynamic and geometric phases can attain the same objective while offering tunable functional control over the polarization of light. We establish a correlation between the structural parameters of metasurface and the topological charge of the resulting vortices. The experimental results fully demonstrate the design’s flexibility and its effective control over the polarization constraints of incident light. Our research uncovers the capacity for vortex generation through the manipulation of hybrid phases introduced by metasurfaces, indicating significant potential for the design of optical devices and the future advancement of innovative optical applications.

## 1 Introduction

Optical vortex beams are typically paraxial beams characterized by their cylindrical symmetric propagation. Notably, the vortex beam’s center is a dark core, where the intensity is nonexistent and remains so throughout its propagation [1], [2]. The wavefront of vortex beams exhibits a spiral-shaped distribution, resulting in its wavevector with an azimuthal component. Consequently, due to the transverse spatial phase distribution, photons acquire orbital angular momentum (OAM) [3], [4], [5]. Owing to these characteristics, vortex beams offer distinct advantages in various fields, including optical trapping, quantum entanglement, nonlinear optics, optical processing, and high-resolution microscopic imaging [2], [6], [7]. In practical applications, vortex beams can be produced directly using active vortex laser generators [8], [9], [10], or more commonly, by utilizing external discrete optical elements to facilitate conversion [11]. However, the optical components utilized in these techniques are bulky and non-planar, presenting challenges for optical integration in various applications.

A metasurface is an artificial material composed of single or multiple sub-wavelength nanostructural units, strategically arranged to perform specific functions [12], [13], [14], [15], [16], [17], [18], [19]. By carefully designing the geometry and composition of the nanostructural unit, one can effectively control the polarization state, amplitude, and phase of a light wave at a sub-wavelength scale [20], [21], [22], [23]. The introduction of metasurfaces has revolutionized the manipulation of light fields through the incorporation of helical wavefronts, whose phases vary with respect to the azimuthal angle. This innovative approach imparts an orbital angular momentum to the beam, effectively transforming it into an optical vortex. By integrating metasurfaces into the design of optical vortex generation devices, it is possible to meet the contemporary demands for miniaturization, portability, and precise polarization control, while also significantly overcoming the limitations associated with traditional vortex-generating methods.

Utilizing metasurfaces, the generation of helical wavefronts is achieved by introducing a spiral spatial phase shift via an array of nanostructural units [24], [25], [26]. As light traverses these nanostructures, it undergoes a phase shift that can be meticulously controlled through design. Theoretically, this phase shift in an optical beam can be dissected into two components: the dynamic phase and the geometric phase [27], [28]. The dynamic phase refers to the phase retardation caused by the optical element as the beam propagates through it. This phase is independent of the polarization or spin states of the light, being mainly influenced by the frequency of the incident light and the refractive index of the material [29]. Given that the structural thickness of nano-units is usually below the operating wavelength, modulation of the dynamic phase is primarily achieved by adjusting the geometric dimensions or spacing of the nano-units, effectively altering the effective refractive index. Conversely, the geometric phase arises from the interaction between spin angular momentum and OAM, showing significant dependence on the polarization state of the incoming light [30], [31]. In the design of metasurfaces, the geometric phase is often induced by rotating anisotropic nano-units, which are specifically tailored for circularly polarized light [26], [32], [33].

The two distinct types of phases – dynamic and geometric – have profoundly influenced the design of numerous metasurfaces for optical vortex generation. Each type offers unique functional advantages grounded in their underlying principles. Despite these innovations, such designs encounter intrinsic limitations, particularly concerning nanoscale structural fabrication precision and stringent polarization control requirements. The dynamic phase and geometric phase exhibit specific correlations with the geometric configuration of the metasurface. By adjusting the dimensions and orientation of nano-units, researchers can create a hybrid phase that not only expands the boundaries of design possibilities but also leads to significant advancements [33], [34], [35], [36], [37], [38]. Since structural modifications affect both phases, achieving precise control over the dimensions of these nano-units is critical for developing effective hybrid phase systems [39]. Therefore, understanding and elucidating the underlying relationships between structural configurations and phase properties are essential steps towards optimizing these designs. This knowledge facilitates the development of more efficient and versatile metasurfaces, capable of overcoming current limitations and pushing the boundaries of optical vortex generation technology.

In this paper, we begin by detailing the methodology for defining and distinguishing the dynamic and geometric phases introduced by metasurfaces. Our approach involves a thorough explanation of how each phase is generated and manipulated through the design of nano-units. Following this, we conduct a quantitative analysis to assess the individual contributions of these phases towards the generation of vortex beams, exploring how their combination can be leveraged to optimize performance. Based on these analyses, we then engineer metasurface structures capable of producing identical vortex beams through diverse design strategies. This step not only demonstrates the flexibility of our approach but also provides a practical validation of our theoretical concepts and design innovations through experimental verification. By comparing theoretical predictions with experimental results, we aim to establish a robust framework for future designs. Moreover, our study investigates the relationship between incident polarization states and various metasurface designs, highlighting their interdependence and implications for optical vortex generation. Through the synergistic integration of geometric and dynamic phases, we showcase the potential for designing metasurfaces that offer enhanced flexibility and augmented functionality. This advancement paves the way for the development of innovative metasurface designs incorporating a range of materials and novel micro-nanostructures, thereby significantly benefiting the performance and capabilities of a multitude of optical devices.

## 2 Results and discussion

### 2.1 Dynamic and geometric phases

**Figure 1:**
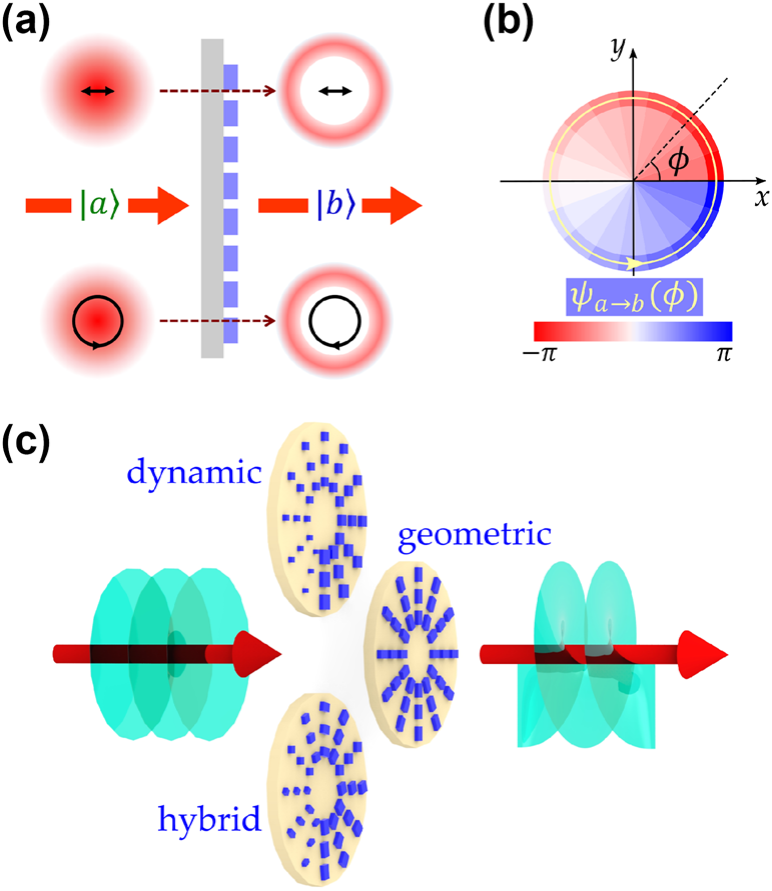
Optical vortex beams generated using metasurfaces. (a) A Gaussian distributed planewave $\left(\left|a\right\rangle \right)$ passes through metasurface chip and is converted into a scalar vortex beam $\left(\left|b\right\rangle \right)$ with doughnut-like intensity distribution. (b) The spiral phase profile is generated by the metasurface for the transformation from $\left|a\right\rangle$ to $\left|b\right\rangle$ . (c) The same transformation from $\left|a\right\rangle$ to $\left|b\right\rangle$ can be accomplished through various distinct designs. Top: generating vortices with dynamic phase gradient by varying the nano-units size of metasurface. Middle: generating vortices with geometric phase gradient by rotating the nano-units orientation for metasurface. Bottom: generating vortices with hybrid phase gradient by adjusting the size and orientation of nano-units.

**Figure 2:**
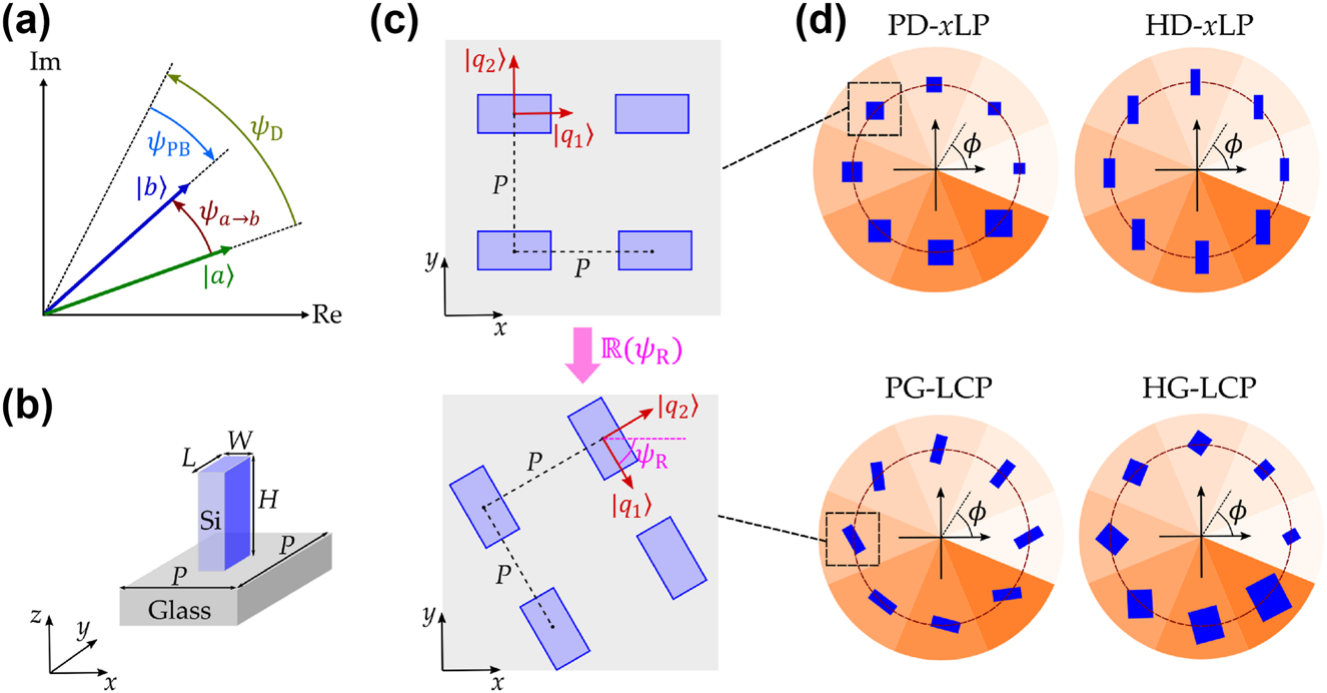
Phase shifts and configurations of metasurface. (a) Phase shifts from $\left|a\right\rangle$ to $\left|b\right\rangle$ induced by metasurface. (b) A nano-unit of the metasurface. (c) A uniform metasurface is achieved utilizing a standard arrangement (upper) and an alternate, rotated arrangement (lower). (d) Four metasurface designs, each based on an eight-sector configuration, are engineered to generate scalar vortex beams. The upper pair of metasurfaces is engineered to convert *x*-polarized light, while the lower pair is designed to transform LCP light.

Firstly, we examine the methodologies for introducing two distinct types of phase shifts. Let $\left|a\right\rangle$ represent the input polarized light state; upon interaction with the metasurface, this light is transformed into the output polarized state $\left|b\right\rangle$ , as illustrated in Figure 1a. In accordance with the Pancharatnam connection, the phase shift between the two states can be indicated as $\psi_{a\to b}=\arg\left(\left\langle a|b\right\rangle \right)$ [28]. We can use Jones matrix J to represent optical elements, so the transformation process can be described as $\left|b\right\rangle =J\left|a\right\rangle$ . Assuming the 2 × 2 matrix J has two orthogonal eigenpolarization states $\left|q_{1}\right\rangle$ , $\left|q_{2}\right\rangle$ and the corresponding eigenvalues *μ*_1_, *μ*_2_. This implies that, for input light with eigenpolarization $\left|q_{1,2}\right\rangle$ , the metasurface imparts a normalized transmittance of $\left|\mu_{1,2}\right|$ and a phase shift of arg(*μ*_1,2_), constituting the eigen-responses. Generally, the phase shift *ψ*_*a*→*b*_ between the input $\left|a\right\rangle$ and the output $\left|b\right\rangle$ can be divided into two components,

(1) $$\psi_{a\to b}=\psi_{\text{D}}+\psi_{\text{PB}},$$

where *ψ*_D_ denotes the dynamic phase and *ψ*_PB_ is named as the geometric phase, also known as the Pancharatnam–Berry (PB) phase [27], [28], [40]. For a lossless conversion, there is *ψ*_D_ = arg(*μ*_1_ *μ*_2_)/2, indicating the expected dynamic phase that the beam acquires upon transmission through the metasurface. Further, *ψ*_PB_ can be given by

(2) $$\psi_{\text{PB}}=\arg\left[\cos\psi_{-}+i\sin\psi_{-}\exp\left(i\psi_{\text{qa}}\right)\right],$$

where $\psi_{-}=\arg\left(\mu_{1}\mu_{2}^{*}\right)/2$ and $\psi_{\text{qa}}=\arg\left(Q\cdot A\right)$ with **Q** and **A** are the Stokes vectors corresponding to $\left|q_{1}\right\rangle$ and $\left|a\right\rangle$ , and the detailed deduction can be found in Section S1 of the Supplementary Information. Owing to the inclusion of the **Q** ⋅ **A** term, *ψ*_PB_ is associated with both the eigenstate of the metasurface and the polarization state of the input light. Figure 2a illustrates this relationship by depicting the phase shifts on the complex plane.

For the generation of an OAM light beam utilizing metasurfaces, a spiral phase shift along the azimuthal angle *ϕ* is necessitated. As shown in Figure 1b, the phase shift $\psi_{a\to b}\left(\phi \right)$ facilitates the generation of a vortex beam, wherein the OAM charge is associated with the gradient ∂*ψ*_*a*→*b*_/∂*ϕ*. According to equation (1), the total OAM charge, denoted as *C*_Tot_, can be bifurcated into two components: the dynamical contribution (*C*_D_ ∝ ∂*ψ*_D_/∂*ϕ*) and the geometrical contribution (*C*_PB_ ∝ ∂*ψ*_PB_/∂*ϕ*), thereby establishing the relationship *C*_Tot_ = *C*_D_ + *C*_PB_ [41], [42]. Using equation (1), we can achieve a phase gradient solely from dynamic phase by maintaining *ψ*_PB_ as a constant value and adjusting arg(*μ*_1_ *μ*_2_). This approach is termed the pure-dynamic design methodology. Alternatively, by sustaining *ψ*_D_ as a constant value, we can accomplish a phase gradient solely from geometric phase. This is achieved by varying *ψ*_PB_ through modulation of $\arg\left(\mu_{1}\mu_{2}^{*}\right)$ and $\arg\left(Q\cdot A\right)$ , as prescribed by equation (2). This methodology for vortex beam generation epitomizes the pure-geometric design approach. In practice, the total phase gradient can be actualized by concurrently modulating *ψ*_D_ and *ψ*_PB_ along *ϕ*, integrating the dynamical and geometrical contributions. This approach, which amalgamates both phase components, can be denoted as the hybrid design methodology. As depicted in Figure 1c, the identical OAM transformation from $\left|a\right\rangle$ to $\left|b\right\rangle$ can be implemented through diverse designs, each characterized by unique properties, particularly regarding their sensitivity to incident polarization states.

### 2.2 General description for lossless metasurfaces

As a fully polarized light beam traverses metasurface structures, the specific nano-unit and its corresponding array induce a definitive phase shift. The configuration of distinctly structured nano-units within an array can induce varying phase shifts. Consequently, a proper design of the nano-unit combined with the strategic arrangement of the array can generate a spiral phase distribution across the transverse plane. For a lossless-transmission metasurface featuring nanofin arrays in the *x* − *y* plane, as shown in Figure 2b and the upper panel of Figure 2c, there generally exist *x*(*y*)-polarized eigenstates denoted by [1,0]^T^ and [0,1]^T^. The corresponding eigenvalues as *μ*_1,2_ = exp(i*φ*_*x*,*y*_), where *φ*_*x*,*y*_ is the introduced phase shift for *x*(*y*)-polarized light by the nano-unit. Moreover, when a rotation angle *ψ*_R_ is imposed on the array (see Figure 2c), the eigenstates associated with the nano-unit will be transformed to $\left|q_{1}\right\rangle =R\left(\psi_{\text{R}}\right){\left[1,0\right]}^{\text{T}}$ and $\left|q_{2}\right\rangle =R\left(\psi_{\text{R}}\right){\left[0,1\right]}^{\text{T}}$ , where R is the two-dimensional rotation matrix. For this rotated nano-unit, the Jones matrix J can be expressed as

(3) $$J=e^{\text{i}\psi_{\text{D}}}\left[\begin{array}{cc} \cos\left(\frac{\psi_{\text{B}}}{2}\right)+i\sin\left(\frac{\psi_{\text{B}}}{2}\right)\cos\left(2\psi_{\text{R}}\right) & i\sin\left(\frac{\psi_{\text{B}}}{2}\right)\sin\left(2\psi_{\text{R}}\right)\\ i\sin\left(\frac{\psi_{\text{B}}}{2}\right)\sin\left(2\psi_{\text{R}}\right) & \cos\left(\frac{\psi_{\text{B}}}{2}\right)-i\sin\left(\frac{\psi_{\text{B}}}{2}\right)\cos\left(2\psi_{\text{R}}\right) \end{array}\right],$$

where *ψ*_D_ = (*φ*_*x*_ + *φ*_*y*_)/2 is the introduced dynamic phase and *ψ*_B_ = *φ*_*x*_ − *φ*_*y*_ denotes the birefringent phase difference between transmissions of the two eigenstates [35], [41]. Building upon the preceding discussion, there are three methodologies to accomplish the spiral phase profile for the generation of scalar vortex beams. In the generations, the dynamic contribution to the OAM charge is associated with ∂*ψ*_D_/∂*ϕ*, whereas the geometric contribution, represented by ∂*ψ*_PB_/∂*ϕ*, is linked to both *ψ*_B_(*ϕ*) and *ψ*_R_(*ϕ*), since *ψ*_−_ = *ψ*_B_/2 and **Q** ⋅ **A** depends on the rotated eigenstates $\left|q_{1}\right\rangle$ .

In the pure-dynamic design approach utilizing metasurfaces, it is essential to maintain the birefringent phase difference at a constant value. A common strategy involves employing structures with circular symmetry, such as cylinders or circular holes [43], to ensure that *ψ*_B_ = 0. For units based on a nanofin structure with *C*_2_ symmetry, it is feasible to fine-tune *μ*_1_ and *μ*_2_ by modulating the dimensions of width *W* and length *L* (see Figure 2b). To achieve *ψ*_B_ = 0, squared nanofins (with *C*_4_ symmetry) are typically employed to induce identical phase shifts in both eigen-responses. Furthermore, by maintaining *ψ*_R_(*ϕ*) as a constant, $\arg\left(Q\cdot A\right)$ becomes fixed, resulting in *C*_PB_ = 0. Subsequently, imposing a spiral profile on *ψ*_D_(*ϕ*) by adjusting *φ*_*x*_ + *φ*_*y*_ of the nano-unit is necessary. The OAM charge of the generated beams is determined solely by *C*_Tot_ = *C*_D_.

For the pure-geometric design approach, by maintaining *ψ*_D_ and *ψ*_−_ fixed as constants, we can manipulate the orientation of the nano-units to modify the value of $\arg\left(Q\cdot A\right)$ , thereby achieving PB-phase metasurfaces. This approach facilitates the generation of phase gradients without altering the dimensions of the nano-units, accomplished solely by adjusting the orientation angle [44], [45], [46]. This technique is straightforward and commonly employed in the design of geometric phase metasurfaces. Specifically, when *ψ*_−_ equals to *π*/2 (i.e., *ψ*_B_ = *π*) and the input light is circularly polarized, the resultant output is also circularly polarized light but with the opposite chirality. In this transformation, a relationship can be derived where *ψ*_*a*→*b*_ = 2*ψ*_R_. This straightforward link facilitates the extensive utilization of circularly polarized light as an ideal input for PB-phase based metasurfaces. For nano-units equipped with a nanofin, specific dimensions can be chosen to set *ψ*_B_ = *π*, followed by rotating the nano-unit to induce a phase gradient along *ϕ*. The resulting OAM charge can be attributed *C*_Tot_ = *C*_PB_ = 2∂*ψ*_R_/∂*ϕ*.

To demonstrate the efficacy of the hybrid design approach, our objective is to achieve an equivalent outcome: generating an identical vortex beam for a specified input light. In this study, we conducted two comparative analyses of hybrid designs against conventional pure-dynamic and pure-geometric designs, respectively. For the pure-dynamic designed metasurface, the total phase gradient ∂*ψ*_*a*→*b*_/∂*ϕ* is solely derived from the dynamic phase profile *ψ*_D_(*ϕ*). For comparative purposes, in the hybrid design, we diminish ∂*ψ*_D_/∂*ϕ* while enhancing ∂*ψ*_PB_/∂*ϕ* to maintain a constant ∂*ψ*_*a*→*b*_/∂*ϕ*. This design requirement can be readily fulfilled by meticulously adjusting *φ*_*x*_ + *φ*_*y*_ and *φ*_*x*_ − *φ*_*y*_ along *ϕ*, while ensuring *ψ*_R_(*ϕ*) = 0. Similarly, for the pure-geometric designed metasurface, the phase gradient ∂*ψ*_*a*→*b*_/∂*ϕ* is entirely produced by the geometric phase profile *ψ*_PB_(*ϕ*). For comparative analysis, we diminish ∂*ψ*_PB_/∂*ϕ* and augment ∂*ψ*_D_/∂*ϕ* to maintain a constant ∂*ψ*_*a*→*b*_/∂*ϕ* in the hybrid design. This manipulation can be accomplished by concurrently adjusting *φ*_*x*_ + *φ*_*y*_ and *ψ*_R_ along *ϕ*, with *φ*_*x*_ − *φ*_*y*_ set to *π*.

### 2.3 Design and simulation

**Figure 3:**
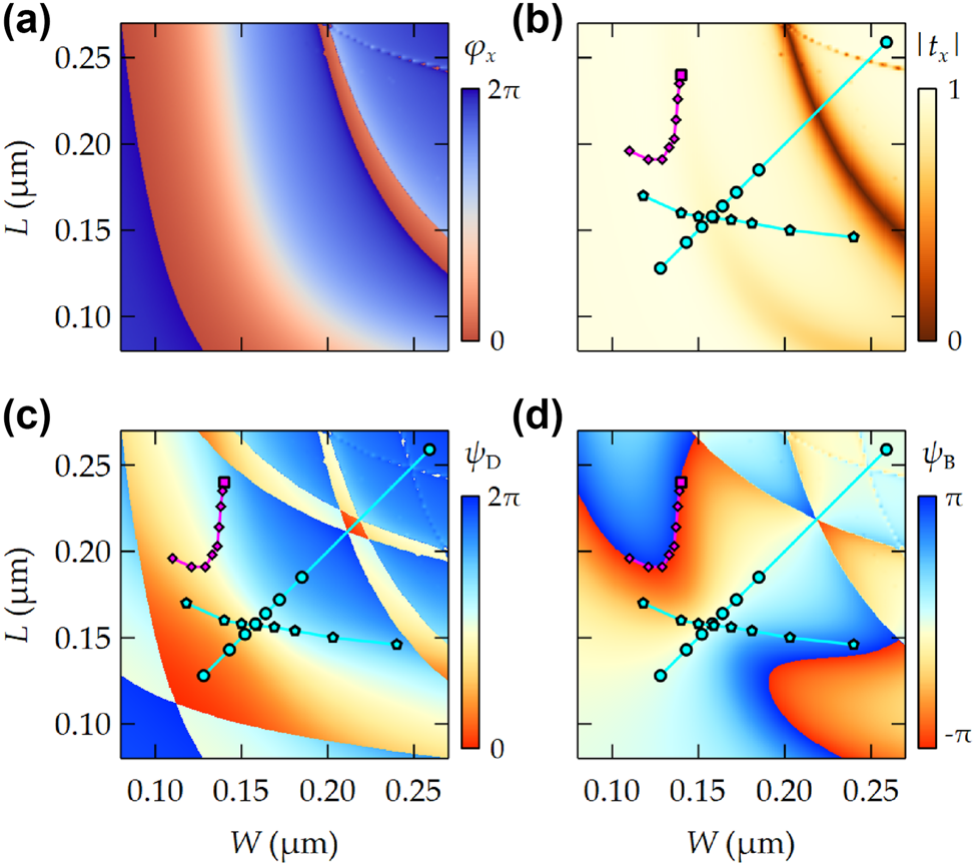
Basic optical features of uniform metasurfaces. (a) The phase shift *φ*_*x*_ and (b) transmittance *t*_*x*_ exhibit dependencies on the nanofin dimensions (*W*, *L*) for *x*-polarized light upon transmission through a uniform metasurface. The *y*-polarized responses can be obtained by exchanging the axes of *W* and *L* in (a) and (b). Upon calculating the phase shifts for *x*-polarized and *y*-polarized lights, (c) the dynamic phase *ψ*_D_ and (d) the birefringent phase difference *ψ*_B_ are determined and illustrated. In (b–d), the eight circular markers correspond to parameters of PD-*x*LP, while the eight pentagonal markers denote parameters of HD-*x*LP. The square marker indicates parameters for PG-LCP, and the eight diamond markers signify parameters for HG-LCP.

In our demonstration, the unit structure consists of a silicon nanofin on a glass substrate, as shown in Figure 2b. To determine the eigen-responses of the nano-unit array, we employ the rigorous coupled-wave analysis (RCWA) to compute the transmitted eigen-responses of periodic array structures upon the incidence of *x*-polarized and *y*-polarized planewaves. In our design and simulation, the pitch of the nano-units was maintained at 400 nm in both directions. With a nanofin height of 400 nm, by varying the width *W* and length *L* of nanofins, the simulated *x*-polarized eigen-responses *φ*_*x*_ and *t*_*x*_ are displayed in Figure 3a and b, respectively. These two parameters respectively denote the phase shift and transmittance experienced by *x*-polarized light upon passing through a uniform array. Due to the *C*_2_ symmetry of the nanofin, the *y*-polarized eigen-responses *φ*_*y*_ and *t*_*y*_ can be easily determined by merely switching the coordinate axes in Figure 3a and b. Utilizing the simulated maps of *φ*_*x*_ and *φ*_*y*_, the corresponding maps for *ψ*_D_ and *ψ*_B_ are derived and displayed in Figure 3c and d, respectively. To generate vortex beams, a spiral spatial phase profile must be incorporated into the cross-section that is orthogonal to the direction of propagation. In our experimental demonstrations, this spiral phase profile is constructed by segmenting the structure into eight sectors along the azimuthal angle *ϕ*, each characterized by discrete phase increments, as shown Figure 2d. Within each sector, a uniform array of nano-units is meticulously arranged to facilitate the desired phase shifts. To approximate a lossless condition, all nano-units are chosen for their high transmittance.

As previously described, we will undertake four metasurface designs to produce scalar vortex beams with an OAM charge of 1. The first design, named PD-*x*LP, is specifically engineered to produce a vortex beam induced solely by a dynamic phase gradient. In this case, the sampled *ψ*_D_(*ϕ*) values for the eight sectors are incrementally increased, while *ψ*_B_(*ϕ*) is set to 0 for all nano-units without any rotation, ensuring that *ψ*_R_(*ϕ*) = 0. In contrast, the hybrid design, termed HD-*x*LP, integrates the spiral phase by sampling both *ψ*_D_(*ϕ*) and *ψ*_B_(*ϕ*), applying incremental steps to the nano-units across the eight sectors, while maintaining *ψ*_R_(*ϕ*) = 0. The schematics for both designs are presented in the first row of Figure 2d. Both the PD-*x*LP and HD-*x*LP designs can convert an *x*-polarized planewave into a scalar vortex beam with an OAM charge of 1. The pure-geometric design, denoted as PG-LCP, is intended for converting a LCP planewave into a vortex beam with the opposite polarization chirality. The gradient of geometric phase is achieved by rotating the uniform nano-units, which have *ψ*_B_(*ϕ*) = *π*, to different angle *ψ*_R_(*ϕ*) within each sector. For comparison, the hybrid design, referred to as HG-LCP, is implemented by incorporating varied *ψ*_D_(*ϕ*) through the adjustment of eight nano-unit structures, while maintaining *ψ*_B_(*ϕ*) = *π*. The illustrations of these two designs are presented in the second row of Figure 2d. Both the PG-LCP and HG-LCP designs have the capability to convert an LCP planewave into an RCP vortex beam with an OAM charge of 1.

**Figure 4:**
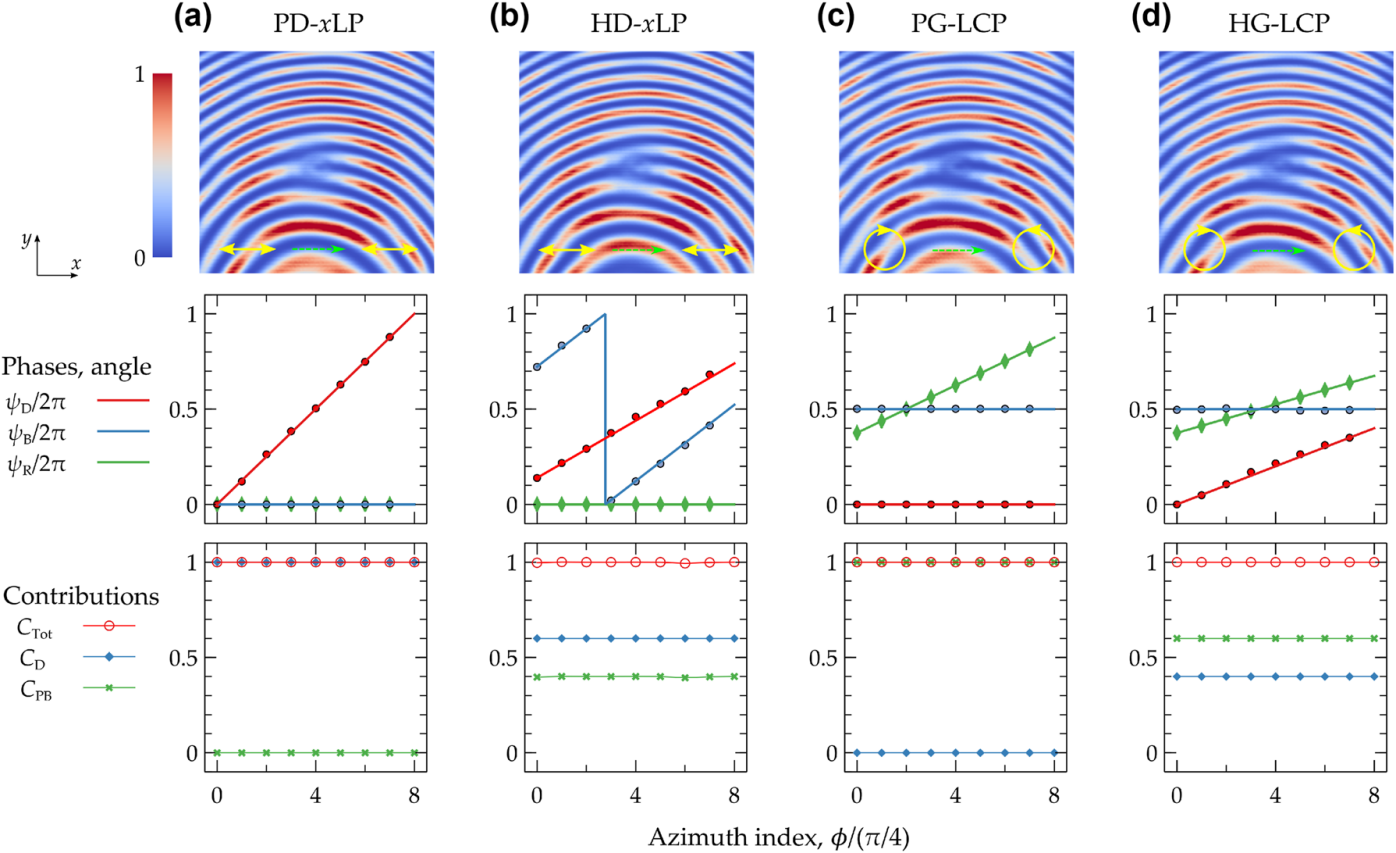
Four distinct metasurface configurations are engineered to produce optical vortex beams. Metasurfaces designed to convert an *x*-polarized plane wave into an *x*-polarized vortex beam using (a) dynamic phase (PD-*x*LP) and (b) hybrid phase (HD-*x*LP). Metasurfaces designed to convert an LCP planewave into an RCP vortex beam using (c) geometric phase (PG-LCP) and (d) hybrid phase (HG-LCP). For (a–d), the first row displays the calculated interference patterns between a conventional Gaussian beam and the generated vortex beam, where there is a shift between the centers of two beams, under the ideal predefined incident polarization. The second row presents the designed phase set {*ψ*_D_, *ψ*_B_, *ψ*_R_} of the nano-unit relative to the azimuth angle *ϕ*. Here, solid lines represent the desired values, while the marked points indicate the actual values of the selected nano-unit in each of the eight sectors. The third row illustrates the contributions of the two types of phase gradients to the OAM charge.

Taking these considerations into account, the dimensions of the nanofins for the four designs (PD-*x*LP, HD-*x*LP, PG-LCP, and HG-LCP) are selected and marked in Figure 3b–d. For each nano-unit within these design, the corresponding set of {*ψ*_D_, *ψ*_B_, *ψ*_R_} is summarized and plotted in the middle panels of Figure 4a–d. Furthermore, the contributions for the OAM charge can be evaluated and displayed in the bottom panels of Figure 4a–d. By utilizing the simulation results of each nano-unit shown in Figure 3a–d, the transformed far field can be calculated with the corresponding input light beams. To confirm the OAM charge of the generated vortices, the simulated interference patterns of the vortex beams with a conventional Gaussian beam are displayed in the top panels of Figure 4a–d. The superposition of a vortex beam and a planewave results in an interference pattern characterized by a fork-like bifurcation at the vortex core [47]. This specific morphology is directly correlated with the OAM charge of the beam. In the patterns shown in Figure 4a–d, a red-colored toroidal contour is a distinctive characteristic of vortex beams, featuring a fork-shaped pattern at the center. This specific pattern includes two distinct branches which signify an OAM charge of 1.

### 2.4 Experimental results

**Figure 5:**
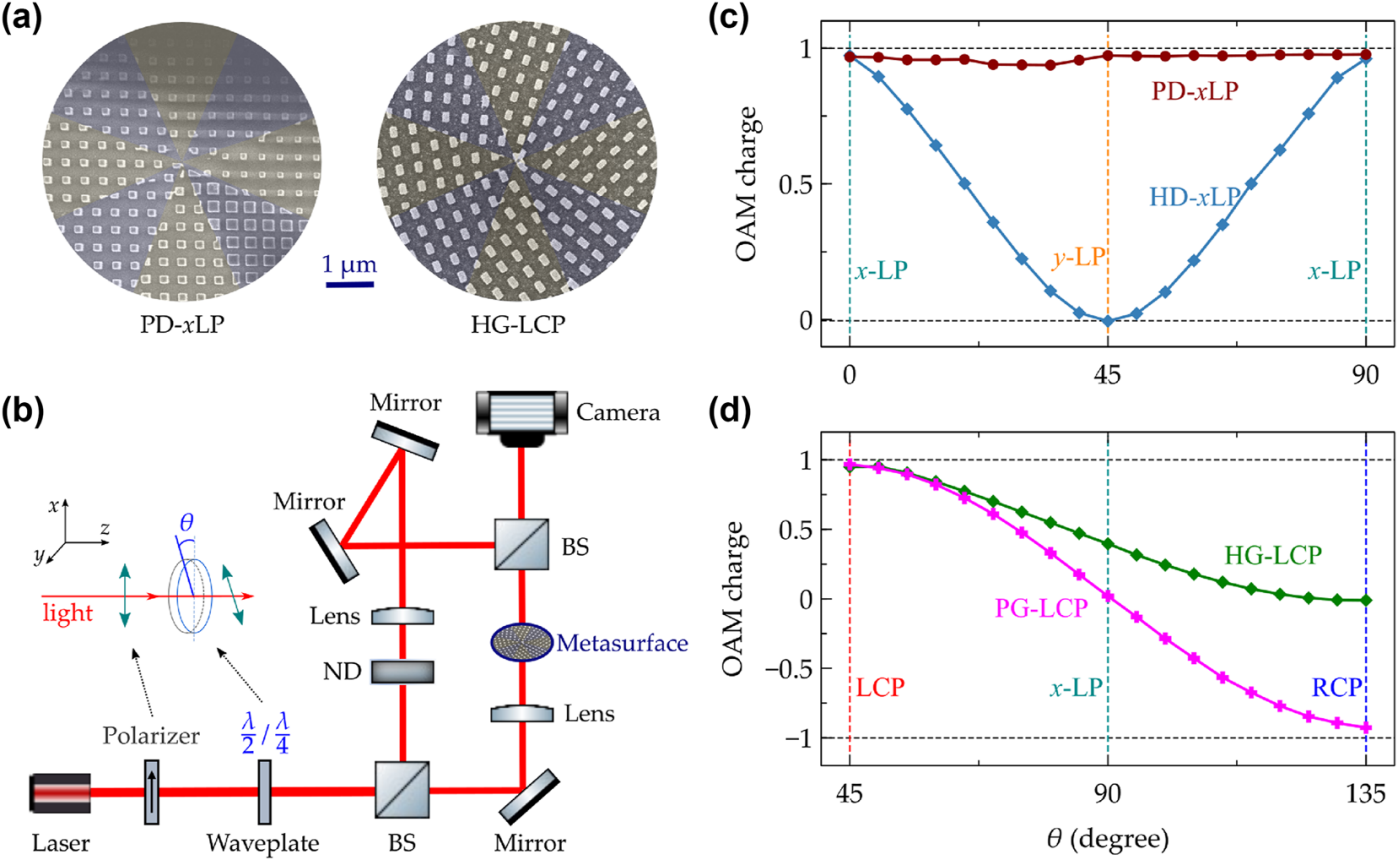
Fabricated metasurfaces and measurement setups. (a) Scanning electron microscope (SEM) images of the fabricated samples of PD-*x*LP and HG-LCP designs. False colors with transparency are used to depict the eight sectors. (b) The proposed measurement apparatus for the optical vortex beams produced by the designed metasurfaces relies on the interference of a reference beam with the generated vortex beam to yield the interference pattern. The beam splitter (BS) facilitates either the division or combination of light beams, while the neutral density filter (ND) is utilized to equilibrate the powers of the two beams, ensuring prominent interference fringes. The inset illustrates a polarizer accompanied by a *λ*/2 (*λ*/4) waveplate, employed to alter the polarization state of the incident light. (c) The average OAM charge of optical vortex beams generated by PD-*x*LP and HD-*x*LP designs is calculated and graphically represented at different directions of incident linear polarizations, which are adjusted using the polarizer and the rotated *λ*/2 waveplate. (d) The average OAM charge of vortex beams produced by PG-LCP and HG-LCP designs is computed and plotted at distinct incident polarization states (from LCP to RCP), which are modified using the polarizer and the rotated *λ*/4 waveplate.

The fabricated samples were created on a borosilicate glass substrate with a thickness of 1 mm, upon which a 400 nm silicon film was deposited. Silicon nanofins were patterned within a circular area of 0.5 mm in diameter using electron-beam lithography followed by a dry etching process. Figure 5a shows scanning electron microscope (SEM) images of two fabricated samples corresponding to the PD-*x*LP and HG-LCP designs. For characterization, a Mach–Zehnder interferometric system was utilized, as depicted in Figure 5b. A laser emitting at a wavelength of 780 nm generates the light beam, which is then polarized in the *x*-direction using a polarizer. A half-wave (*λ*/2) or quarter-wave (*λ*/4) waveplate is subsequently employed to convert the *x*-polarized light into linearly, elliptically, or circularly polarized light. The beam is split into two paths, with one passing through the metasurface chip. The two beams are then recombined, creating interference patterns that are recorded by a charge-coupled device (CCD) camera. As illustrated in the inset of Figure 5b, adjusting the angle *θ* between the optical axes of the polarizer and the *λ*/2 (or *λ*/4) waveplate allows for the generation of any desired linearly or circularly polarized light. By leveraging these polarization modifications, we confirm the creation of vortex beams facilitated by the metasurfaces and further evaluate the dependence of various metasurfaces on the polarization states of the incident light.

**Figure 6:**
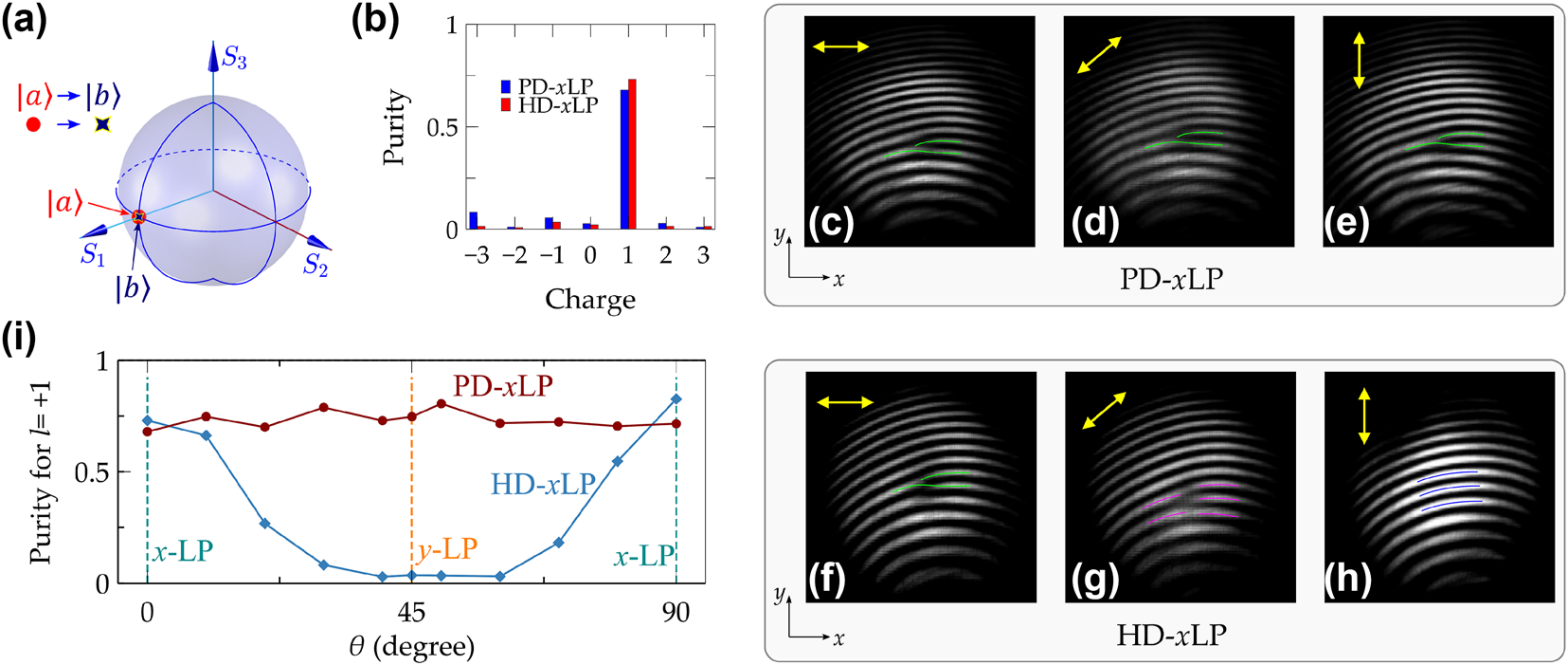
Transformation of an *x*-polarized planewave into an *x*-polarized vortex beam with an OAM charge of 1, utilizing two distinct designs: PD-*x*LP and HD-*x*LP. (a) The polarization states of both input and output beams are represented on the Poincaré sphere. (b) The experimental charge purity of this transformation for both designed metasurfaces under *x*-polarized incident light. Measured interference patterns for optical vortex beams generated by PD-*x*LP and HD-*x*LP metasurfaces at different directions of incident linear polarizations: (c, f) *x*-polarized, (d, g) 45° linearly polarized, and (e, h) *y*-polarized. (i) The purity of the topological charge of 1 within vortex beams generated by the two designs under various incident linear polarizations. In (c–h), to clearly indicate the bifurcation status, including the branch numbers and directions, green, magenta, and blue lines are employed to guide the bright fringes.

The designed metasurfaces of PD-*x*LP and HD-*x*LP are engineered to convert an *x*-polarized plane wave into a vortex beam with an OAM charge of 1 while maintaining *x*-polarization. As illustrated in Figure 5c and schematically depicted in Figure 6a, these designs aim at generating vortex beams with specific OAM charges from linearly polarized light. The measured charge purity for *x*-polarized incident light is displayed in Figure 6b (refer also to Section S4 of the Supplementary Information for additional details). The highest purity for an OAM charge of 1 is observed with magnitudes of 0.68 for PD-*x*LP and 0.73 for HD-*x*LP. These values indicate the efficiency of converting the input plane wave into a vortex beam with the desired OAM charge. The suboptimal charge purity primarily results from low-resolution phase steps across the eight sectors and dislocations at the boundaries. These imperfections can lead to deviations from the ideal phase profile required for generating vortex beams with high purity. Moreover, the effect of incident polarization states on the generation of vortex beams is clarified. In configurations involving a polarizer and a *λ*/2 waveplate, the orientation of linear polarization rotates by 2*θ* corresponding to the rotation *θ* of the *λ*/2 waveplate. This setup allows for the adjustment of the polarization state of the incident light. For the pure-dynamic metasurface (PD-*x*LP), the rotation of the *λ*/2 waveplate exerts minimal impact on the outcomes. This is consistent with its polarization-independent properties, as shown in Figure 5c. It means that the light field and OAM charge of the generated vortex beams remain unaffected by the polarization state of the input light. The interference fringes displayed in Figure 6c–e demonstrate this characteristic, showing that the vortex beams generated by PD-*x*LP maintain their integrity regardless of the input polarization state. These patterns confirm the robustness of the PD-*x*LP design against variations in the polarization of the incident light. In the case of the hybrid design (HD-*x*LP), there is a clear dependence on the polarization state of the incident light, as illustrated by the calculated OAM charge in Figure 5c. This design produces an optimal vortex beam with an OAM charge of 1 when the incident light is *x*-polarized. However, if the incident light is *y*-polarized, the output does not form a vortex beam but remains a planewave, manifesting as alternating bright and dark stripes in the interference fringes. These observations are supported by experimental results presented in Figure 6f–h (refer to Section S3 for further details in the Supplementary Information).

It is indeed crucial to highlight that both the PD-*x*LP and HD-*x*LP designs are capable of producing identical vortex beams when using *x*-polarized incident light. This capability underscores their functionality under optimal polarization conditions. However, their performance varies significantly with changes in the polarization state of the incident light. To further assess how sensitive these designs are to different incident polarizations, the purity of the topological charge 1 within the generated vortex beams was measured and illustrated in Figure 6i. The findings clearly indicate the differing responses of the pure-dynamic metasurface (PD-*x*LP) and the hybrid design (HD-*x*LP) to variations in the polarization state of incident light. This design of PD-*x*LP exhibits robustness against changes in the incident polarization. Regardless of the polarization state of the incoming light, PD-*x*LP maintains its functionality in generating vortex beams with an OAM charge of 1. The purity of the generated vortex beams remains high, showing that this design is less sensitive to the polarization state of the incident light. This characteristic makes PD-*x*LP particularly suitable for applications where the polarization of the light source might fluctuate or cannot be precisely controlled. In contrast, the HD-*x*LP shows a significant dependence on the polarization state of the incident light. While it performs optimally under *x*-polarized light, producing vortex beams with high purity, its performance deteriorates when the polarization state changes. This behavior is consistent with theoretical predictions shown in Figure 5c, which indicate the hybrid nature’s reliance on specific polarization states for optimal operation. These results emphasize the importance of considering the application environment and the level of control over polarization states when choosing between PD-*x*LP and HD-*x*LP designs. Understanding these differences allows for the selection of the most appropriate metasurface based on the requirements for polarization stability and adaptability.

**Figure 7:**
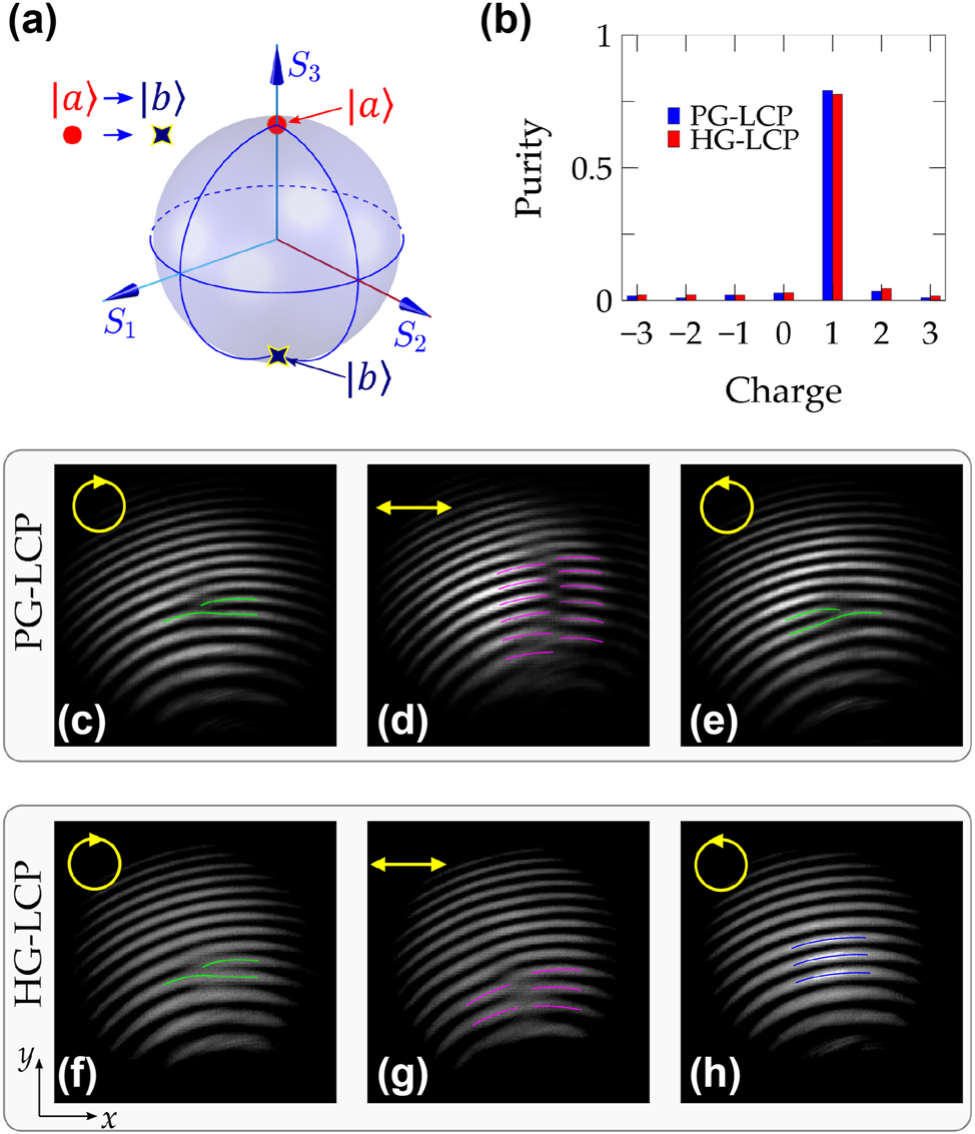
Transformation of an LCP planewave into an RCP vortex beam carrying an OAM charge of 1, utilizing two distinct designs: PG-LCP and HG-LCP. (a) The polarization states of both input and output beams are represented on the Poincaré sphere. (b) The experimental charge purity of this transformation for both designed metasurfaces under LCP incident light. Measured interference patterns for optical vortex beams generated by PG-LCP and HG-LCP metasurfaces at different incident polarization states: (c, f) LCP, (d, g) *x*-polarization, and (e, h) RCP. In (c–h), to clearly indicate the bifurcation status, including the branch numbers and directions, green, magenta, and blue lines are employed to guide the bright fringes.

As shown in Figure 5d and schematically illustrated in Figure 7a, the metasurfaces labeled PG-LCP and HG-LCP are designed to transform an LCP planewave into an RCP vortex beam carrying an OAM charge of 1. The measured charge purity for LCP incident light is depicted in Figure 7b, with additional details available in Section S4 of the Supplementary Information. The highest purity for an OAM charge of 1 is observed at magnitudes of 0.79 for PG-LCP and 0.78 for HG-LCP. These values indicate the efficiency and effectiveness of these designs in converting the input polarization state into the desired output vortex beam with a specific OAM charge. Typically, the geometric phase introduced by metasurfaces is highly sensitive to the polarization state of the incident light. As the light passes through the polarizer and the *λ*/4 waveplate, its polarization can be adjusted to linear, elliptical, or circular polarization based on the rotation angle *θ* of the *λ*/4 waveplate. This adjustment capability allows for fine-tuning of the input light’s polarization state before it interacts with the metasurface. According to calculations illustrated in Figure 5d, the OAM charge generated by the pure-geometric design (PG-LCP) is highly dependent on the polarization state of the incident light. Specifically, the sign of the OAM charge is inverted for input light of opposite chirality (i.e., switching from LCP to RCP light). This phenomenon reflects the conjugate symmetry inherent in the relationship between the input and output fields, meaning that the handedness of the vortex beam is directly related to the handedness of the incident light. From the interference fringes shown in Figure 7c and e, it is evident that input light of distinct chiralities (LCP vs RCP) leads to the formation of interference fringes oriented in opposite directions. This indicates a direct relationship between the handedness of the incident light and the resulting vortex beam’s characteristics. However, when the input light is linearly polarized, the output light undergoes transformation into a vector vortex beam (see Section S3 of the Supplementary Information). Upon interference with circularly polarized reference light, an interlaced pattern of interference fringes is produced, both theoretically and experimentally as shown in Figure 7d. For the hybrid design (HG-LCP), the OAM charge of the output light remains associated with the incident polarization state (see Figure 5d). However, compared to the pure-geometric design (PG-LCP), the fluctuation in the OAM charge is less pronounced, and no distinct reversal of the OAM charge sign is observed when switching the chirality of the input light. Experimental findings indicate that a well-defined vortex beam is generated when the input light has LCP chirality (see Figure 7f). Conversely, the use of RCP light yields output light with a negligible topological charge, leading to an interference pattern marked by alternating bright and dark stripes (see Figure 7h).

As a result, both PG-LCP and HG-LCP metasurfaces are capable of generating identical vortex beams from LCP input light. However, their reliance on the incident polarization state differs. The PG-LCP design is highly sensitive to the polarization state, functioning optimally only under specific conditions (i.e., LCP input). Conversely, the HG-LCP metasurface, attributed to its dynamic characteristics, diminishes this dependence on polarization. It provides enhanced stability across a range of polarization states while still maintaining optimal performance with LCP input. Consequently, these attributes make the HG-LCP design potentially more adaptable for applications where variations in the polarization state of the incident light are expected. In contrast, the PG-LCP design may be preferred in scenarios where precise control over the polarization state is feasible.

## 3 Discussion and conclusion

In this study, we demonstrate that metasurfaces with diverse designs can generate scalar vortex beams. Compared to previous works [19], [31], [33], [35], [37], several key differences and advancements are noted:

– Focus on Design Multiplicity: Unlike earlier research, our investigation centers on the variety of methodologies and design configurations that enable achieving identical OAM transformations. This exploration underscores the multiplicity of approaches available to achieve a singular objective. Notably, hybrid designs offer solutions to certain limitations related to material selection and form factors for nano-units.
– Hybrid Design Integration: Our hybrid design enables the modulation of both dynamic and geometric phases within a single metasurface chip. By fine-tuning the dimensions of meta-atoms and the array configuration, we can precisely control the contributions of dynamic and geometric phases to the OAM charge. Each contributed charge can be fractional, while ensuring that the total charge remains an integer. Moreover, our approach allows for the geometric phase to be designed and imposed not only for typical circularly polarized lights but also for other polarization states.
– Control of Polarization Sensitivity: Transformations based on hybrid designs present distinct advantages. Pure-dynamic designs lack selectivity towards the polarization state of input light. In contrast, hybrid designs can incorporate a polarization filter, with sensitivity or bandwidth customizable by adjusting the geometric contribution. Pure-geometric designs impose strict requirements on the polarization state of the input light; however, these constraints can be mitigated in hybrid designs through the integration of dynamic contributions. The sensitivity observed during the OAM transformation process is influenced by and can be controlled through the relative contributions of dynamic and geometric phases.
– Quantitative Assessment: By utilizing equation (3) and calculations from our previous work [41], we quantitatively determine the contributions of both dynamic and geometric phases. This allows for a comprehensive assessment of sensitivity, facilitating a direct correlation with specific design parameters of the transformation. Such an approach not only enhances our understanding of the underlying mechanisms but also paves the way for more flexible and robust metasurface designs.

The investigation of input sensitivity extends beyond a single polarization state or scalar depiction. It encompasses distinct dynamic or geometric contributions at various azimuthal positions within the spatial distribution of light fields. While the overall transformation contribution remains constant, different azimuthal locations exhibit varying degrees of polarization sensitivity. This variability is particularly useful for studying the evolution of certain spatial beam propagations. Furthermore, we can engineer specific contribution ratios for OAM transformations across different radial zones on the metasurface. Even when the specified OAM transformation is uniform, deviations in input polarization lead to varied polarization evolution characteristics along the radial direction. Consequently, this produces a diverse array of vector light fields. Detailed descriptions and simulations supporting these findings are provided in Sections S5–7 of the Supplementary Information, offering deeper insights into the mechanisms underlying these phenomena and illustrating potential applications.

In our experiment, we successfully generated a vortex beam carrying an OAM charge of 1. Importantly, there are no inherent constraints preventing the production of higher-order optical vortices using hybrid designs; this can be achieved by increasing the number of sampling sectors and enhancing the requisite phase gradient. Beyond scalar vortex beams, hybrid designs also offer the capability to produce vector vortex beams, thanks to the incorporated geometric contributions. However, as indicated in Figures 6b and 7b, the charge purity was found to be suboptimal, largely due to the coarse resolution of the phase steps. To address this issue, stepped phase transitions can be mitigated by implementing quasi-continuous variations in the designed phase along the azimuthal angle. This involves continuously adjusting the dimensions of the nanofins according to their phase maps (refer to Figure 3). Additionally, adopting a variable pitch instead of a fixed one can enhance the homogeneity of transmission. Integrating machine learning techniques with our composite dynamic-geometric phase design principles offers another promising avenue for refining the phase profile, resulting in smoother transitions. By leveraging machine learning’s predictive power and pattern recognition capabilities, we can optimize the phase design process, ensuring more seamless and continuous phase distributions across the vortex beam’s azimuthal angle. This approach not only improves the quality of the generated beams but also broadens the applicability of our design rules with composite phases in various optical systems.

The measured transmittance of the fabricated metasurfaces is approximately 70 %, which is primarily attributed to reflections at the air-glass interface and the non-negligible absorption by silicon nanofins at the wavelength of 780 nm. To improve this transmittance, antireflection film can be deposited on the substrate to significantly reduce reflections at the air–glass interface. Additionally, alternative materials such as silicon nitride or lithium niobate could be considered for their lower absorption characteristics at the operational wavelength, thereby enhancing the overall performance. An important consideration is that structural resonance has a more significant impact on the low transmission of nano-units compared to material absorption. Therefore, in the design of transmission, it is crucial to select dimensions for the nano-units that are far from structural resonances.

Metasurfaces are capable of generating both dynamic and geometric phases, which have been widely utilized to produce optical vortex beams. However, by leveraging the combined dynamic and geometric phases, a broader range of innovative and diverse functionalities can be realized in the engineering of optical vortex beam generation. In this study, we achieve a spiral phase distribution through the customization of hybrid phases, thereby producing scalar vortex beams. Moreover, the contributions of orbital angular momentum (OAM) from the two phases can be precisely allocated. By employing metasurfaces that incorporate hybrid phases, we introduce an optimization strategy for the engineering of nano-units, addressing both structural and functional dimensions. The hybrid design concept is not restricted by the material, structure, or propagation manner of metasurfaces. This principle opens up new possibilities for employing hybrid phases to engineer a variety of phase profiles, which can facilitate novel functionalities and pave the way for future innovations in optical devices.

## Supplementary Material

Supplementary Material Details

## References

[1] Dennis M. R., O’Holleran K., Padgett M. J. (2009). Singular optics: optical vortices and polarization singularities. *Prog. Opt.*.

[2] Shen Y. (2019). Optical vortices 30 years on: OAM manipulation from topological charge to multiple singularities. *Light: Sci. Appl.*.

[3] Molina-Terriza G., Torres J. P., Torner L. (2007). Twisted photons. *Nat. Phys.*.

[4] Franke-Arnold S., Allen L., Padgett M. J. (2008). Advances in optical angular momentum. *Laser Photonics Rev.*.

[5] Bliokh K. Y., Nori F. (2015). Transverse and longitudinal angular momenta of light. *Phys. Rep.*.

[6] Yao A. M., Padgett M. J. (2011). Orbital angular momentum: origins, behavior and applications. *Adv. Opt. Photonics*.

[7] Qing C., Cui J., Feng L., Zhang D. (2024). Thermal atoms facilitate intensity clipping between vectorial dual-beam generated by a single metasurface chip. ..

[8] Miao P. (2016). Orbital angular momentum microlaser. *Science*.

[9] Zhang Z. (2020). Tunable topological charge vortex microlaser. *Science*.

[10] Sroor H. (2020). High-purity orbital angular momentum states from a visible metasurface laser. *Nat. Photonics*.

[11] Wang X., Nie Z., Liang Y., Wang J., Li T., Jia B. (2018). Recent advances on optical vortex generation. *Nanophotonics*.

[12] Yu N. (2011). Light propagation with phase discontinuities: generalized laws of reflection and refraction. *Science*.

[13] Meinzer N., Barnes W. L., Hooper I. R. (2014). Plasmonic meta-atoms and metasurfaces. *Nat. Photonics*.

[14] Kamali S. M., Arbabi E., Arbabi A., Faraon A. (2018). A review of dielectric optical metasurfaces for wavefront control. *Nanophotonics*.

[15] Zhu Y., Zang X., Chi H., Zhou Y., Zhu Y., Zhuang S. (2023). Metasurfaces designed by a bidirectional deep neural network and iterative algorithm for generating quantitative field distributions. *Light: Adv. Manuf.*.

[16] Chi H. (2024). Metasurface enabled multi-target and multi-wavelength diffraction neural networks. *Laser Photonics Rev.*.

[17] Xiong B. (2023). Breaking the limitation of polarization multiplexing in optical metasurfaces with engineered noise. *Science*.

[18] Fan Q. (2018). Broadband generation of photonic spin-controlled arbitrary accelerating light beams in the visible. *Nano Lett.*.

[19] Liu M. (2021). Broadband generation of perfect Poincaré beams via dielectric spin-multiplexed metasurface. *Nat. Commun.*.

[20] Yu N., Capasso F. (2014). Flat optics with designer metasurfaces. *Nat. Mater.*.

[21] Overvig A. C. (2019). Dielectric metasurfaces for complete and independent control of the optical amplitude and phase. *Light: Sci. Appl.*.

[22] Bai Y., Lv H., Fu X., Yang Y. (2022). Vortex beam: generation and detection of orbital angular momentum [invited]. *Chin. Opt. Lett.*.

[23] Zhou Y. (2024). Directional phase and polarization manipulation using Janus metasurfaces. *Adv. Sci.*.

[24] Zhang Y., Liu W., Gao J., Yang X. (2018). Generating focused 3D perfect vortex beams by plasmonic metasurfaces. *Adv. Opt. Mater.*.

[25] Wang D. (2020). High-efficiency metadevices for bifunctional generations of vectorial optical fields. *Nanophotonics*.

[26] Guo Y. (2022). Classical and generalized geometric phase in electromagnetic metasurfaces. *Photonics Insights*.

[27] Berry M. (1987). The adiabatic phase and Pancharatnam’s phase for polarized light. *J. Mod. Opt.*.

[28] Gutiérrez-Vega J. C. (2011). Pancharatnam-Berry phase of optical systems. *Opt. Lett.*.

[29] Khorasaninejad M. (2016). Polarization-insensitive metalenses at visible wavelengths. *Nano Lett.*.

[30] Bliokh K. Y., Rodríguez-Fortuño F. J., Nori F., Zayats A. V. (2015). Spin-orbit interactions of light. *Nat. Photonics*.

[31] Devlin R. C., Ambrosio A., Rubin N. A., Mueller J. P. B., Capasso F. (2017). Arbitrary spin-to-orbital angular momentum conversion of light. *Science*.

[32] Marrucci L., Manzo C., Paparo D. (2006). Optical spin-to-orbital angular momentum conversion in inhomogeneous anisotropic media. *Phys. Rev. Lett.*.

[33] Wang E. W., Phan T., Yu S.-J., Dhuey S., Fan J. A. (2022). Dynamic circular birefringence response with fractured geometric phase metasurface systems. *Proc. Natl. Acad. Sci.*.

[34] Arbabi A., Horie Y., Bagheri M., Faraon A. (2015). Dielectric metasurfaces for complete control of phase and polarization with subwavelength spatial resolution and high transmission. *Nat. Nanotechnol.*.

[35] Balthasar Mueller J., Rubin N. A., Devlin R. C., Groever B., Capasso F. (2017). Metasurface polarization optics: independent phase control of arbitrary orthogonal states of polarization. *Phys. Rev. Lett.*.

[36] Zhang S. (2021). Dynamic display of full-Stokes vectorial holography based on metasurfaces. *ACS Photonics*.

[37] Zhou J., Liu Y., Ke Y., Luo H., Wen S. (2015). Generation of Airy vortex and Airy vector beams based on the modulation of dynamic and geometric phases. *Opt. Lett.*.

[38] Lv H., Bai Y., Yao J., Yang Y., Ma X., Stratakis E., Luo X., Hong M., Pu M., Li X. (2021). Generation of optical vortices using the metasurface combining dynamic and geometric phases. *Proc. SPIE 12072, 10th International Symposium on Advanced Optical Manufacturing and Testing Technologies: Micro- and Nano-Optics, Catenary Optics, and Subwavelength Electromagnetics*.

[39] Wang Z. (2022). Bifunctional manipulation of terahertz waves with high-efficiency transmissive dielectric metasurfaces. *Adv. Sci.*.

[40] Martínez-Fuentes J. L., Albero J., Moreno I. (2012). Analysis of optical polarization modulation systems through the Pancharatnam connection. *Opt. Commun.*.

[41] Zhang D., Feng X., Huang Y. (2018). Orbital angular momentum induced by nonabsorbing optical elements through space-variant polarization-state manipulations. *Phys. Rev. A*.

[42] Zhang D., Feng X., Cui K., Liu F., Huang Y. (2015). Identifying orbital angular momentum of vectorial vortices with Pancharatnam phase and Stokes parameters. *Sci. Rep.*.

[43] Sun J., Wang X., Xu T., Kudyshev Z. A., Cartwright A. N., Litchinitser N. M. (2014). Spinning light on the nanoscale. *Nano Lett.*.

[44] Chen M., Jiang L., Sha W. (2018). Orbital angular momentum generation and detection by geometric-phase based metasurfaces. *Appl. Sci.*.

[45] Huo P. (2020). Photonic spin-multiplexing metasurface for switchable spiral phase contrast imaging. *Nano Lett.*.

[46] Karimi E., Schulz S. A., De Leon I., Qassim H., Upham J., Boyd R. W. (2014). Generating optical orbital angular momentum at visible wavelengths using a plasmonic metasurface. *Light: Sci. Appl.*.

[47] Liu G.-G. (2018). Measurement of the topological charge and index of vortex vector optical fields with a space-variant half-wave plate. *Opt. Lett.*.

