# Supplementary material for: Exploiting the combined dynamic and geometric phases for optical vortex beam generation using metasurfaces
Source: Nanophotonics. 2025 Mar 11;14(5):635–46. doi: 10.1515/nanoph-2025-0004 (PMC11953722; doi:10.1515/nanoph-2025-0004)
Supplement: Supplementary file 1 — Supplementary Material Details [file j_nanoph-2025-0004_suppl_001.pdf]

Supplementary Information: Exploiting the combined dynamic and geometric phases for optical vortex beam generation using metasurfaces

*Jialong Cui, Chen Qing, Lishuang Feng, and Dengke Zhang**

School of Instrumentation and Optoelectronic Engineering, Beihang University, Beijing 100191, China

dkzhang@buaa.edu.cn

S1. Phase shift induced by a nano-unit of metasurfaces

Consider $|a\rangle$ as the input polarized light. By utilizing a metasurface equipped with the Jones matrix $\mathbb{J}$ for the nano-unit, we can derive the output polarized light $|b\rangle = \mathbb{J}\,|a\rangle$. The phase shift between two states introduced in this conversion can be expressed as

$$\psi_{a\to b} = \arg\left[\mu_1 + \mu_2 + (\mu_1 - \mu_2)\mathbf{Q}\cdot\mathbf{A}\right], \tag{S1}$$

where $\mu_{1,2}$ and $|q_{1,2}\rangle$ are the eigenvalues and eigenstates of the Jones matrix $\mathbb{J}$, $\mathbf{Q}$ and $\mathbf{A}$ are the Stokes vectors corresponding to the the eigenstate $|q_1\rangle$ and the input state$|a\rangle$, respectively [1, 2]. For lossless nano units, the modulus of $\mu_{1,2}$ equates to 1. Consequently, $\mu_{1,2}$ can be represented as $\exp(\mathrm{i}\varphi_{1,2})$, where $\varphi_{1,2}$ represents the phase shift corresponding to the eigenstate $|q_{1,2}\rangle$ when it is the input light. The resultant product of $\mathbf{Q}\cdot\mathbf{A}$ is likewise a unit length complex number, hence the term $\mathbf{Q}\cdot\mathbf{A}$ can also be expressed by $\exp(\mathrm{i}\psi_{\mathrm{qa}})$, where $\psi_{\mathrm{qa}} = \arg(\mathbf{Q}\cdot\mathbf{A})$. Therefore, equation (S1) can be represented in complex form as

$$\psi_{a\to b} = \arg\left[\mathrm{e}^{\mathrm{i}\varphi_1} + \mathrm{e}^{\mathrm{i}\varphi_2} + (\mathrm{e}^{\mathrm{i}\varphi_1} - \mathrm{e}^{\mathrm{i}\varphi_2})\mathrm{e}^{\mathrm{i}\psi_{\mathrm{qa}}}\right]. \tag{S2}$$

Let $\psi_{\mathrm{D}} = (\varphi_1 + \varphi_2)/2$ and $\psi_- = (\varphi_1 - \varphi_2)/2$ and equation (S2) can be deduced as

$$\psi_{a\to b} = \arg\left\{\mathrm{e}^{\mathrm{i}\psi_{\mathrm{D}}}\left[\mathrm{e}^{\mathrm{i}\psi_-} + \mathrm{e}^{-\mathrm{i}\psi_-} + (\mathrm{e}^{\mathrm{i}\psi_-} - \mathrm{e}^{-\mathrm{i}\psi_-})\mathrm{e}^{\mathrm{i}\psi_{\mathrm{qa}}}\right]\right\}. \tag{S3}$$

Here, we redefine $\psi_{a\to b}$ by incorporating a phase variable, ψ_{PB}, as

$$\psi_{a\to b} = \arg\left(\mathrm{e}^{\mathrm{i}\psi_{\mathrm{D}}}\mathrm{e}^{\mathrm{i}\psi_{\mathrm{PB}}}\right), \tag{S4}$$

comparing with equation (S3), there is

$$\mathrm{e}^{\mathrm{i}\psi_{\mathrm{PB}}} = \mathrm{e}^{i\psi_-} + \mathrm{e}^{-\mathrm{i}\psi_-} + (\mathrm{e}^{\mathrm{i}\psi_-} - \mathrm{e}^{-\mathrm{i}\psi_-})\mathrm{e}^{\mathrm{i}\psi_{\mathrm{qa}}}. \tag{S5}$$

According to equation (S4), we can simply divide the phase shift $\psi_{a\to b}$ into two components ψ_{D} and ψ_{PB}, that is

$$\psi_{a\to b} = \psi_{\mathrm{D}} + \psi_{\mathrm{PB}}. \tag{S6}$$

In equation (S6), ψ_{D} represents the common phase delay for two eigen-responses and remains independent of polarization states, termed as the dynamic phase. The phase ψ_{PB} corresponds specifically to the geometric phase, also known as the Pancharatnam-Berry (PB) phase. Using equation (S5), the PB phase can be readily obtained

$$\psi_{\mathrm{PB}} = \arg[\cos\psi_- + \mathrm{i}\sin\psi_- \mathrm{e}^{\mathrm{i}\psi_{\mathrm{qa}}}]. \tag{S7}$$

Regarding equation (S7), two particular scenarios arise for the PB phase: firstly, when $\psi_- = \pi/2 + k\pi$, where k is an integer, ψ_{PB} equals ψ_{qa}; and secondly, when $\psi_{\mathrm{qa}} = 0$, ψ_{PB} is equivalent to ψ_-. These two distinctive conditions offer us distinct methodologies for the quantitative manipulation of the geometric phase.

In our four designs, the required parameters $\{\psi_{\mathrm{D}}, \psi_{\mathrm{B}}, \psi_{\mathrm{R}}\}$ are summarized in Figures 3 and 4 of the main text. The corresponding dimensions of the nanofins for each design are detailed in Tables S1-S4.

Tab. S1: Designed geometric parameters of meta-atoms in PD-xLP

Meta-atom	Azimuth index, $\phi/(\pi/4)$							
	0	1	2	3	4	5	6	7
W (nm)	128	143	152	158	164	172	185	259
L (nm)	128	143	152	158	164	172	185	259
ψ_{R} (deg)	0	0	0	0	0	0	0	0

Tab. S2: Designed geometric parameters of meta-atoms in HD-xLP

Meta-atom	Azimuth index, $\phi/(\pi/4)$							
	0	1	2	3	4	5	6	7
W (nm)	118	140	150	159	169	181	203	240
L (nm)	170	160	158	157	156	154	150	146
ψ_{R} (deg)	0	0	0	0	0	0	0	0

Tab. S3: Designed geometric parameters of meta-atoms in PG-LCP

Meta-atom	Azimuth index, $\phi/(\pi/4)$							
	0	1	2	3	4	5	6	7
W (nm)	140	140	140	140	140	140	140	140
L (nm)	240	240	240	240	240	240	240	240
ψ_{R} (deg)	135.125	157.625	180.125	202.625	225.125	247.625	270.125	292.625

Tab. S4: Designed geometric parameters of meta-atoms in HG-LCP

Meta-atom	Azimuth index, $\phi/(\pi/4)$							
	0	1	2	3	4	5	6	7
W (nm)	110	121	129	133	136	137	138	139
L (nm)	196	191	191	198	203	214	226	235
ψ_{R} (deg)	135	148.5	162	175.5	189	202.5	216	229.5

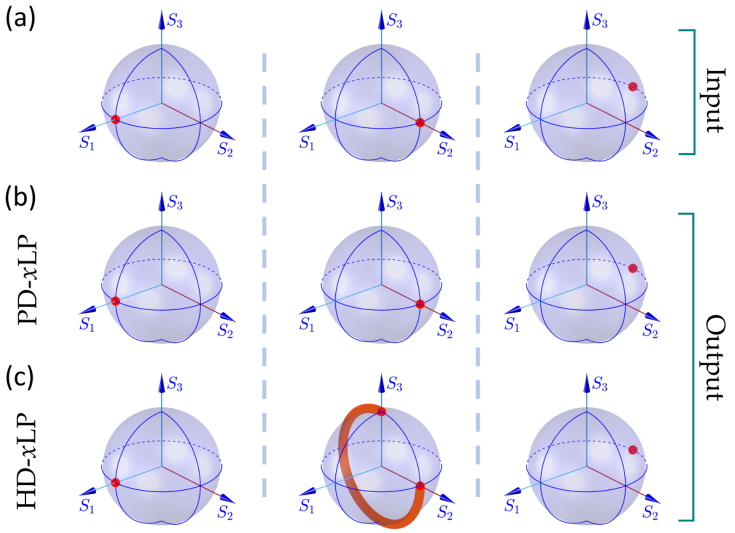

Fig. S1: The SOP distributions of both input and output beams are represented on the Poincaré sphere. (a) Three different SOPs of the input beam: x-polarized light (left), 45°linearly polarized light (center), and y-polarized light (right). (b) The resultant SOP distribution of the converted beams induced by the pure-dynamic design PD-xLP. (c) The resultant SOP distribution of the converted beams induced by the hybrid design HD-xLP.

S2. The state of polarization involved in the conversion

The pure-dynamic metasurface imparts a zero phase difference between the two eigenpolarizations. Consequently, in the ideal case where the input light is a scalar beam, the output beam retains its scalar nature. The various states of polarization (SOP) of the input light, as illustrated in the Poincaré sphere shown in Fig. S1a, the corresponding output states of PD-xLP are displayed in Fig. S1b. As anticipated, there is no discernible difference between the SOPs of the input and output lights. In the experiment, maintaining zero phase differences and equal transmittance for two eigen-responses proves challenging. This difficulty can lead to subtle variations in the polarization state of the output light. In the case of hybrid design metasurfaces, which also incorporate a geometric phase, the nonzero birefringent phase difference leads to alterations in the SOP of the output light. Consequently, apart from the two eigenpolarization inputs, other polarized lights are transformed into vector beams. The SOP distribution of generated output lights of HD-xLP are shown in Fig. S1c. It is important to note that the OAM charges remain consistent for both PD-xLP and HD-xLP when the incoming light is x-polarized. However, the OAM charges differ when the incoming light is y-polarized, as demonstrated by the calculations presented in Figure 5(c) in the main text.

For the metasurfaces of both the pure-geometric design PG-LCP and the hybrid design HG-LCP, a consistent birefringent phase difference of π is established for the two eigen-states. This functions as a half-wave plate, capable of orthogonally transforming circularly polarized light. For input light possessing other SOPs, the transformation procedure for each unit remains analogous, attributed to the identical birefringent phase difference. However, owing to the rotation of the nano units along the azimuthal angle ϕ, the resulting output light manifests as a vector beam. As shown in Figs. S2a-c, the LCP input light is transformed into RCP light for both PG-LCP and HG-LCP designs. Conversely, the RCP light is converted to LCP light. However, when the input light is linearly x-polarized, the output beam becomes a vector beam. It should be noted that while the converted SOP distributions exhibit similarities for both the PG-LCP and the HG-LCP designs, the OAM charges of the resultant beams differ, with the exception of the LCP input light. This distinction is illustrated in Figure 5(d) of the main text.

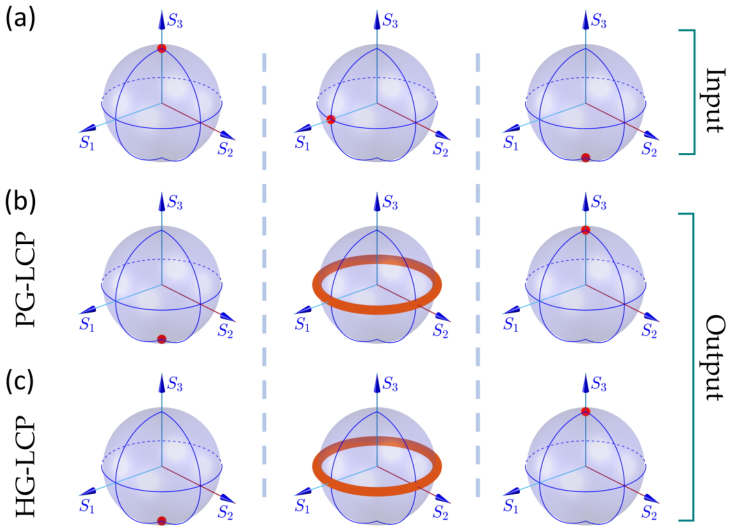

Fig. S2: The SOP distributions of both input and output beams are represented on the Poincaré sphere. (a) Three different SOPs of the input beam: LCP light (left), x-polarized light (center), and RCP light (right). (b) The resultant SOP distribution of the converted beams induced by the pure-geometric design PG-LCP. (c) The resultant SOP distribution of the converted beams induced by the hybrid design HG-LCP.

S3. Far-field intensity and interference fringe

As depicted in Figure 5(b) of the main text, the SOP of the input light can be precisely controlled by adjusting the angle between the optical axes of the polarizer and the wave plate within the measurement setup. In the experiments, the interference patterns produced by the interaction between the generated vortex beam and a reference beam of varying polarization inputs are captured using a CCD camera. Additionally, the far-field intensity distribution of the vortex beams can be easily determined by blocking the reference light. The measurement and simulated outcomes for the four designs are presented in Figs. S3a,b-S4a,b for input lights with varying SOP. By comparing with the calcualted OAM charge curves displayed in Figures 5(c) and 5(d) of the main text, we observed that the far-field intensity and interference pattern of the output light align with the behavior exhibited by the calculated OAM charge of vortex beams.

S4. OAM purity for the fabricated metasurfaces

To evaluate the OAM charges of the generated vortex beams, the fundamental approach is to expand the generated field using

$$E(\phi) = \sum A_n \exp(in\phi), \tag{S8}$$

From equation (S8), any Gaussian-distributed scalar field with a topological charge n (where $n \neq 0$) corresponds to a vortex field, which results in a dark spot at the beam center. If a spiral phase with a charge of $-n$ is applied to such a field, it will be transformed into a plane wave with a Gaussian-like distribution, featuring a bright spot at the center. Thus, by altering the charge of the applied spiral phase, it is possible to screen vortex fields with different topological charges, and the purity ($\propto A_n^2$) can be evaluated by measuring the magnitude of the central spot. In the experiment, the application of different spiral phases can be achieved using a spatial light modulator (SLM), with the corresponding setup as shown in Fig. S5a. The $\lambda/2$ ($\lambda/4$) waveplate placed in front of the metasurface serve to

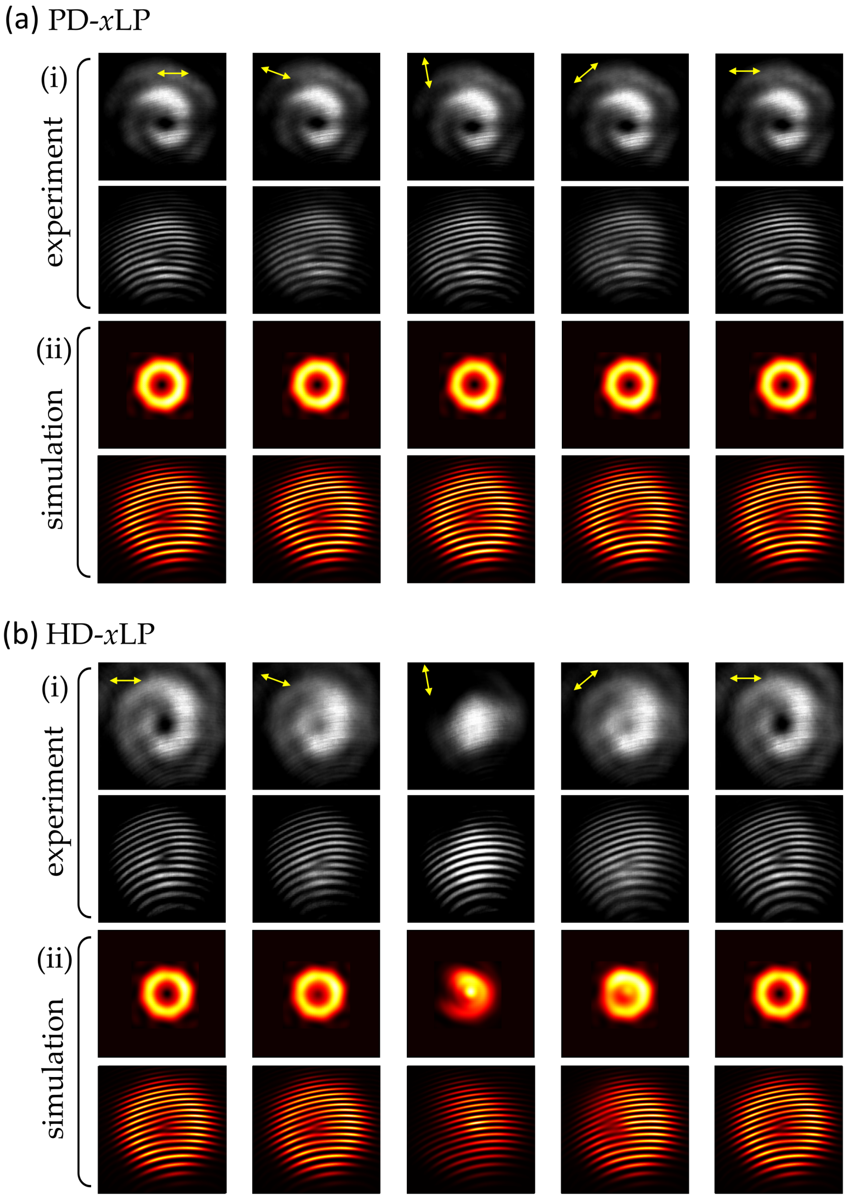

Fig. S3: The far-field pattern of the generated vortex beam, along with the corresponding interference fringes produced by varied linearly polarized lights traversing the metasurfaces. The results of vortex beams generated by two designs are presented: (a) pure-dynamic design (PD-xLP) and (b) hybird design (HD-xLP). For each design, both (i) experimental and (ii) simulation results are displayed. In (i) and (ii), the top panel displays the far-field patterns as the polarization direction of the input linearly polarized light rotates from x-polarized, transitions to y-polarized, and then returns to x-polarized. The bottom panel is the interference fringes corresponding to the top panel. The yellow arrow highlighted in the top-left corner denotes the SOP of input light.

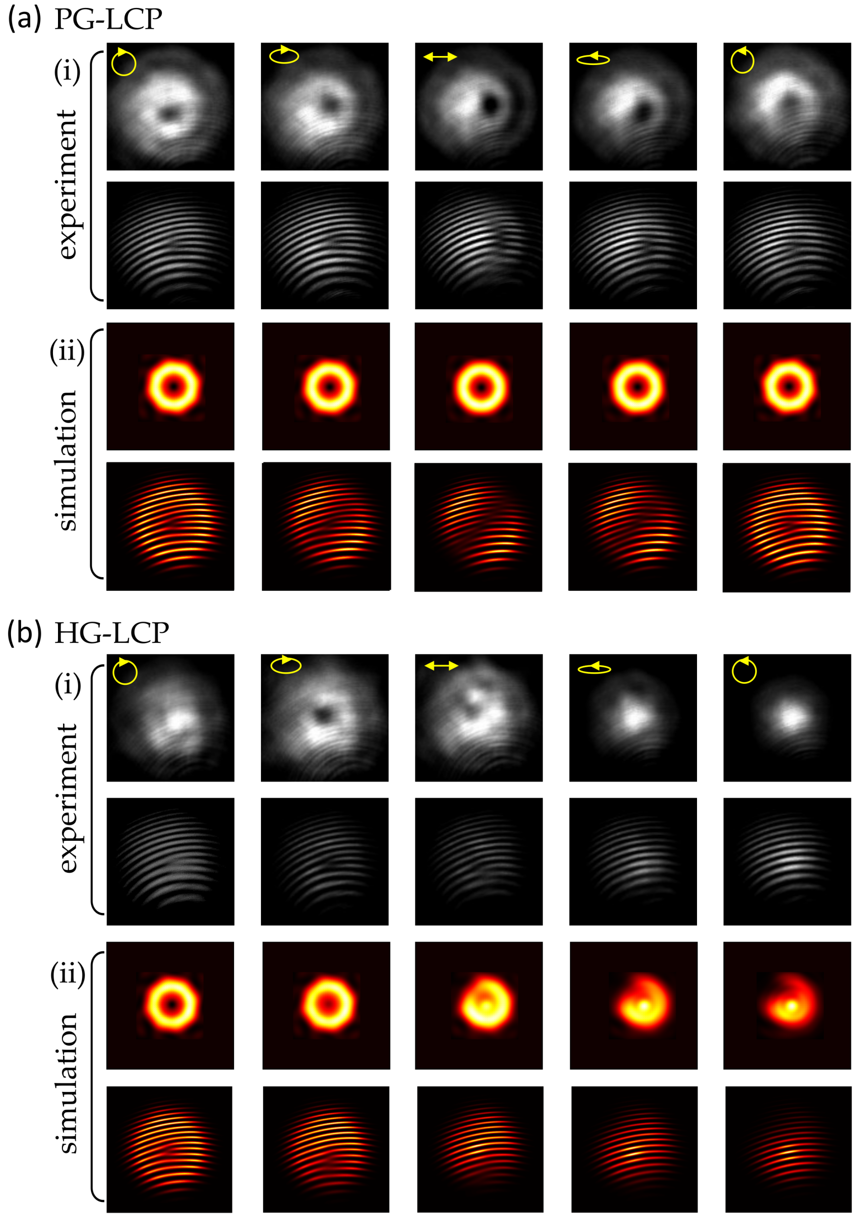

Fig. S4: The far-field pattern of the generated vortex beam, along with the corresponding interference fringes produced by varied polarized lights traversing the metasurfaces. The results of vortex beams generated by two designs are presented: (a) pure-geometric design (PG-LCP) and (b) hybird design (HG-LCP). For each design, both (i) experimental and (ii) simulation results are displayed. In (i) and (ii), the top panel exhibits the far-field patterns as the SOP of the input light shifts from LCP, transforms to x-polarization, and subsequently changes to RCP. The bottom panel is the interference fringes corresponding to the top panel. The yellow circle with arrow highlighted in the top-left corner denotes the SOP of input light.

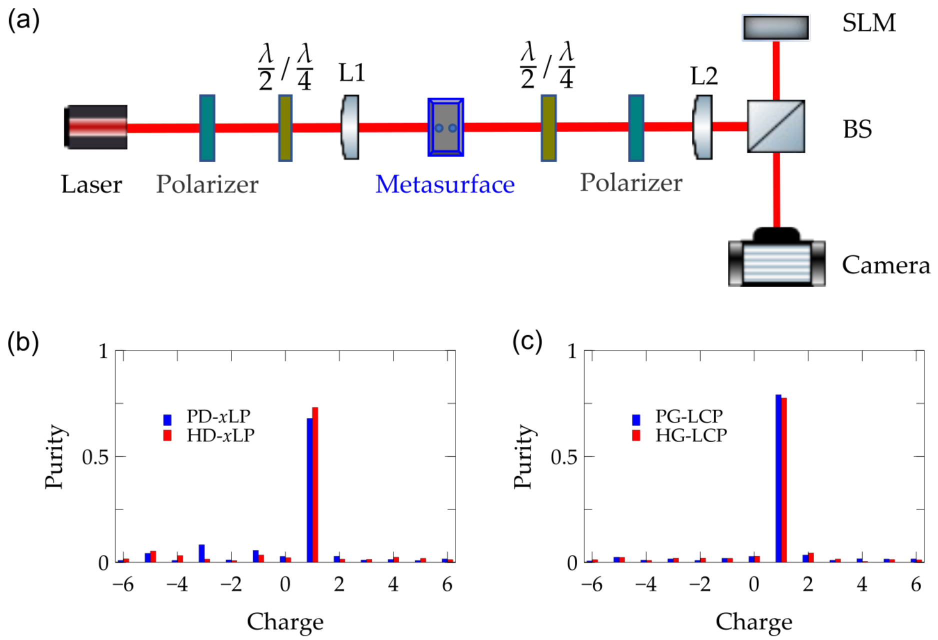

Fig. S5: (a) The measurement setup for characterizing the purity of OAM charges of the generated vortex beams with metasurfaces. (b) The purity for charges from −6 to 6 with the designs of PD-xLP and HD-xLP. (c) The purity for charges from −6 to 6 with the designs of PG-LCP and HG-LCP.

adjust the polarization of the input light, while the $\lambda/2$ ($\lambda/4$) waveplate behind the metasurface are used to alter the polarization of the generated light to the required polarization for the SLM. The measured purity for charges ranging from −6 to 6 is presented in Figs. S5b,c. Figure S5b specifically illustrates the results for the two designed metasurfaces aimed at transforming an x-polarized plane wave into an x-polarized vortex beam with an OAM charge of 1. Figure S5c presents the results of the two designed metasurfaces for the transformation of an LCP plane wave into an RCP vortex beam with an OAM charge of 1. These results are also depicted in Figures 6(b) and 7(b) of the main text. To elucidate the dependence of the generated vortex beams on the input polarization, we configured the charge of the spiral phase to be 1 using the SLM and assessed the purity across various incident polarization states. The outcomes of this assessment are illustrated in Figure 6(i) of the main text.

S5. Polarization dependence of the demonstrated metasurfaces

In equation (S6), we bifurcate the phase shift of the metasurface into two distinct components and provide a quantitative description under specified conditions. This analytical framework not only provides the design of metasurfaces with tailored phase gradients for generating vortex beams adaptable to any polarization state but also facilitates the prediction of additional metasurface characteristics. Regarding equation (S7), the geometric phase is intimately linked to the polarization state of the incident light. As a result, alterations in the incident light's polarization state will influence the output. Hence, fluctuations in the OAM charges can be utilized as an indicator of the metasurface's sensitivity to polarization.

Figure S6a illustrates the conversion of an x-polarized plane wave into an x-polarized vortex beam carrying an OAM charge of 1. Figure S6b presents the OAM charges generated by various polarization inputs passing through two distinct metasurface configurations: PD-xLP and HD-xLP. In the pure-dynamic phase metasurface, variations in the polarization state do not influence the OAM charge, aligning with experimental observations. Conversely, in the hybrid design, the OAM charge is sensitive to the incident polarization due to contributions from geometric phases. Figure S6c illustrates the conversion of an LCP plane wave into an RCP vortex beam with an OAM charge

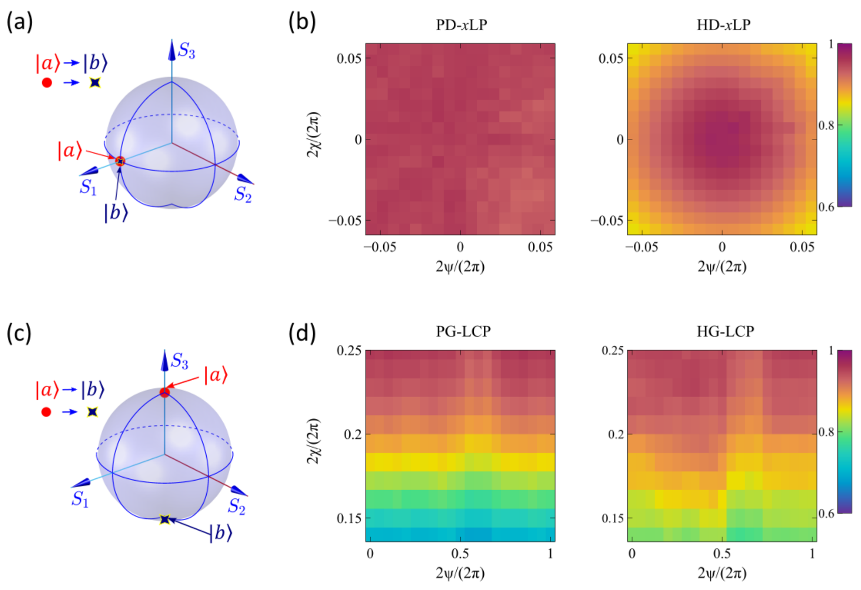

Fig. S6: Polarization sensitivity of the metasurfaces demonstrated in the main text. (a) Transformation of an x-polarized plane wave into an x-polarized vortex beam with an OAM charge of 1. The polarization states of both input and output beams are represented on the Poincaré sphere. (b) For the transformation in (a), the OAM charge of the generated vortex beam is calculated under varying incident polarization states for two designs: PD-xLP and HD-xLP. (c) Transformation of an LCP plane wave into an RCP vortex beam carrying an OAM charge of 1. The polarization states of both input and output beams are represented on the Poincaré sphere. (d) For the transformation in (c), the OAM charge of the generated vortex beam is calculated under varying incident polarization states for two designs: PG-LCP and HG-LCP. In (b) and (d), the axes of 2ψ and 2χ correspond to the azimuth angle and zenith angle, respectively, on the Poincaré sphere.

of 1. Figure S6d demonstrates that the OAM charge varies for different polarization inputs when passing through the two distinct metasurfaces of PG-LCP and HG-LCP. The OAM charge varies across both designs; however, its dependency diverges based on the proportion of geometric contributions relative to the overall total.

S6. Metasurface design with uniform phase gradients

Figure S7a illustrates the conversion of an x-polarized plane wave into an x-polarized vortex beam possessing an OAM charge of 1. Figure S7b presents four distinct designs aimed at accomplishing the transformation depicted in Fig. S7a. While each design incorporates unique dynamic and geometric features, they all maintain a consistent gradient along the ϕ axis. Figure S7c illustrates the corresponding variation in OAM charge for different incident polarizations. It is evident that the sensitivity of OAM charge to polarization can be engineered by adjusting the ratio of geometric to dynamic contributions, with higher sensitivity achieved for a larger proportion of geometric contributions.

S7. Metasurface design with non-uniform phase gradients

Figure S8a illustrates the conversion of an x-polarized plane wave into an x-polarized vortex beam possessing an OAM charge of 2. Figure S8b presents three distinct designs aimed at accomplishing the transformation depicted in Fig. S8a. Each design integrates distinct dynamic and geometric features, accompanied by variations in the phase gradient along the ϕ axis. Figure S8c depicts the resultant changes in OAM charge for diverse incident polarizations.

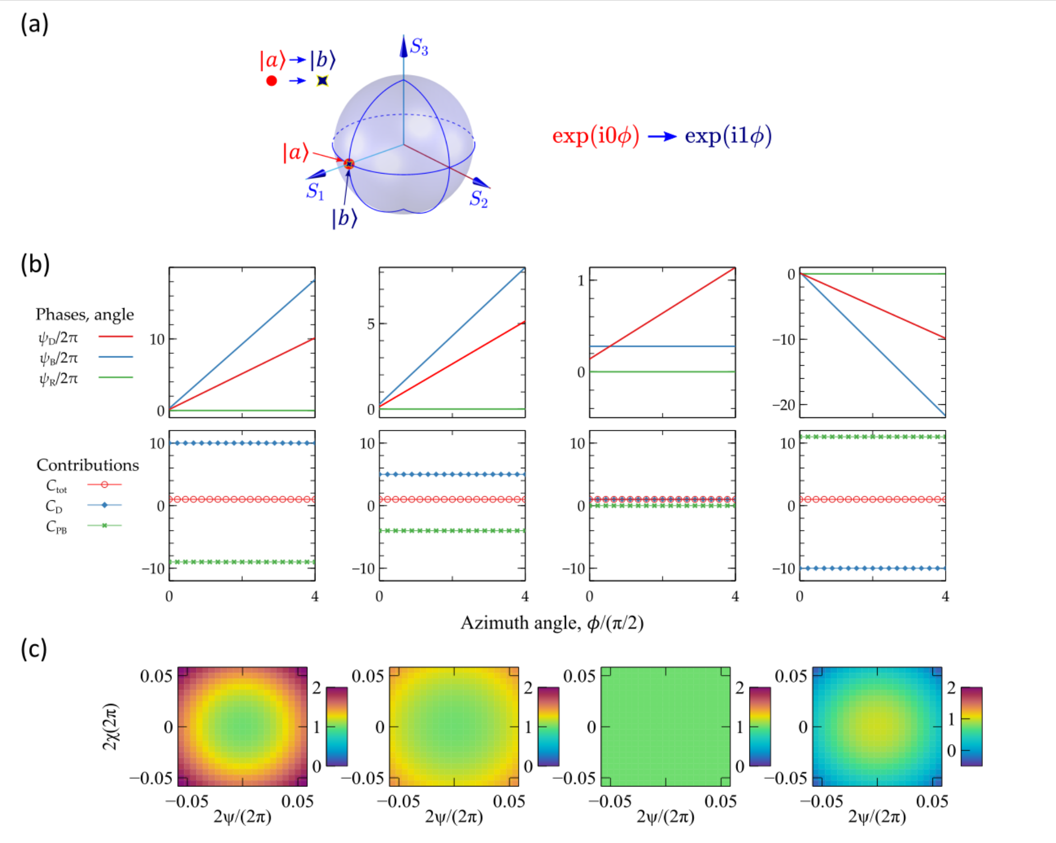

Fig. S7: (a) Transformation of an x-polarized plane wave into an x-polarized vortex beam with an OAM charge of 2. (b) Four distinct designs aimed at accomplishing the transformation depicted in (a). (c) The sensitivity of OAM charge to the input polarization for the designs in (b).

Comparing the first two results in Fig. S8c, it is evident that the sensitivity of OAM charge to polarization can be maintained across different designs with non-uniform phase gradients. Additionally, the sensitivity can also be engineered using non-uniform phase gradient approaches, as demonstrated by the last result in Fig. S8c.

References

[1] Gutiérrez-Vega, J. C. Pancharatnam-berry phase of optical systems. *Opt. Lett.* **36**, 1143 (2011).

[2] Zhang, D., Feng, X. & Huang, Y. Orbital angular momentum induced by nonabsorbing optical elements through space-variant polarization-state manipulations. *Phys. Rev. A* **98**, 043845 (2018).

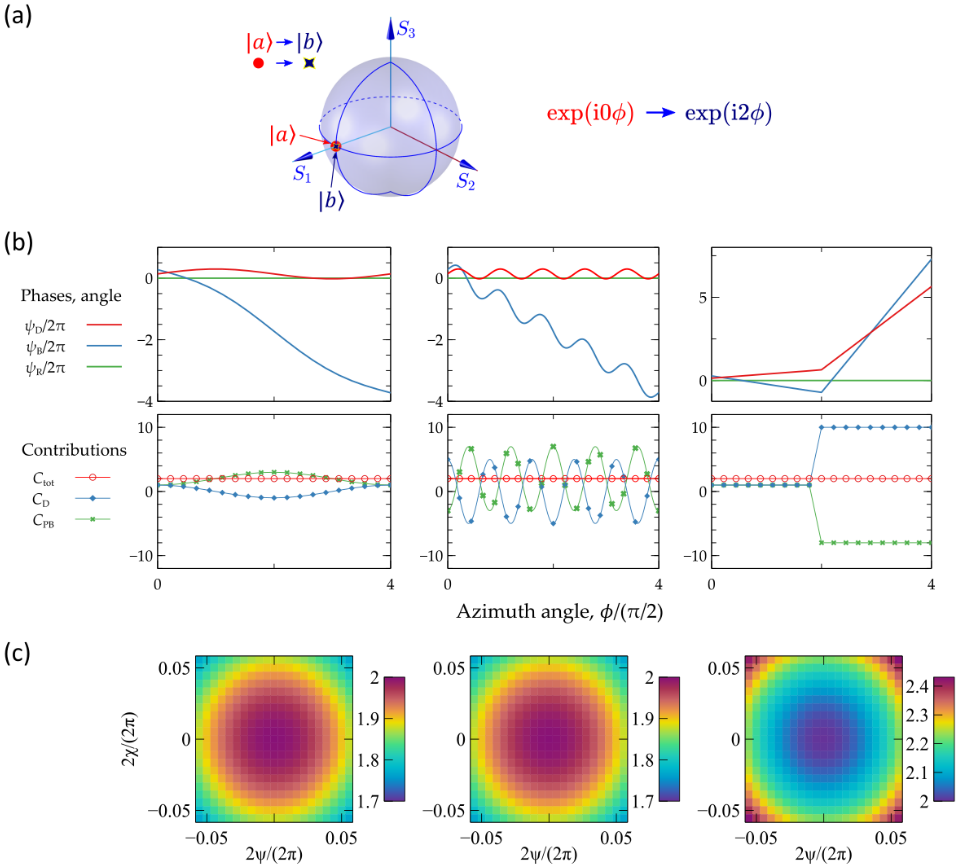

Fig. S8: (a) Transformation of an x-polarized plane wave into an x-polarized vortex beam with an OAM charge of 2. (b) Three distinct designs aimed at accomplishing the transformation depicted in (a). (c) The sensitivity of OAM charge to the input polarization for the designs in (b).
